# Four new lignans from the leaves and stems of *Schisandra propinqua* var. *sinensis*

**DOI:** 10.1007/s13659-013-0017-8

**Published:** 2013-04-03

**Authors:** Shan-Zhai Shang, Ying-Shan Han, Yi-Ming Shi, Xue Du, Cheng-Qin Liang, Mark A. Wainberg, Zhong-Hua Gao, Wei-Lie Xiao, Han-Dong Sun

**Affiliations:** 117 State Key Laboratory of Phytochemistry and Plant Resources in West China, Kunming Institute of Botany, Chinese Academy of Sciences, Kunming, 650201 China; 217 McGill University AIDS Centre, Lady Davis for Medical Research, Jewish General Hospital, Montreal, Quebec, Canada; 317 University of Chinese Academy of Sciences, Beijing, 100049 China

**Keywords:** *Schisandra propinqua* var. *sinensi*, schpropinrins, lignan, anti-human immunodeficiency virus integrase activity

## Abstract

Four new tetrahydrofuran lignans, schpropinrins A-D (**1–4**), together with five known ones, were isolated from the leaves and stems of *Schisandra propinqua* var. *sinensis*. Their structures, including absolute configurations, were characterized by means of spectroscopic analysis and ECD calculation. Compounds **1–4** featured a ketal or hemiketal substructure at C-7 and all of the isolates were tested for their anti-HIV integrase activity.

**Electronic Supplementary Material:**

Supplementary material is available for this article at 10.1007/s13659-013-0017-8 and is accessible for authorized users.

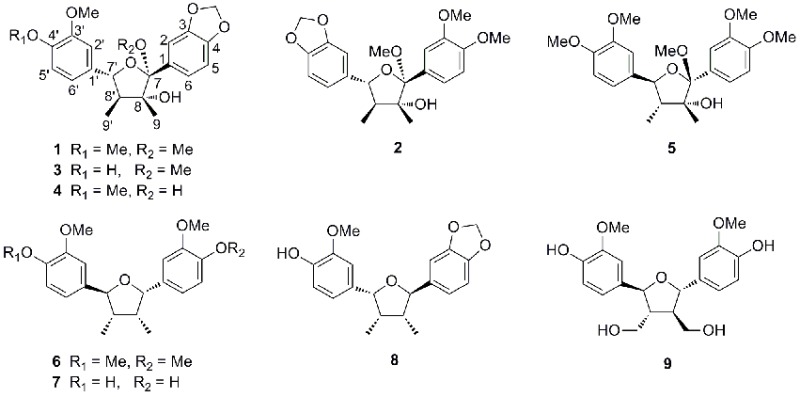

## Introduction

Phytochemical investigations on species of the family Schisandraceae have revealed that they are rich sources of lignans, which possessed various beneficial pharmacological effects, such as antitumor,[1] cytotoxic,[2–4] anti-HIV,[5,6] antioxidative,[7,8] antihepatitis,[9] and hepatoprotective effects.[1]

*Schisandra propinqua* var. *sinensis*, popularly known as "tie-gu-san" in the Shennongjia district of mainland China, is used as folk medicine for the treatment of arthritis, traumatic injury, gastralgia, angeitis, and other related diseases.[10] Aiming at the discovery of biologically active natural products, phytochemical research on this plant was carried out. As a result, four new tetrahydrofuran lignans, schpropinrins A–D (**1**–**4**), together with five known ones, were isolated and their absolute structures were elucidated by extensive spectroscopic studies and quantum chemical calculation (Fig. 1). The five known tetrahydrofuran lignans were identified to be henricine A (**5**),[11] ganschisandrin (**6**),[12] 2*R*-(2*α*,3*α*,4*α*,5*β*)-4,4'-(tetrahydro-3,4-dimethyl-2,5-furandiyl)bis(2-methoxy-phenol) (**7**),[13] chicanine (**8**),[14] neoolivil (**9**).[15] All of the compounds were evaluated in an anti-HIV integrase DNA binding assay.

## Results and Discussion

Schpropinrin A (**1**) was obtained as yellow oil and its

**Figure 1.** Structures of compounds **1**–**9**

molecular formula of $C_{22}H_{26}O_7$ was established from HREIMS ($[M]^+$, *m/z* 402.1688) and $^{13}C$ NMR (DEPT) spectroscopic data, indicating 10 degrees of unsaturation. The IR spectrum showed the presence of hydroxyl group (3443 $cm^{-1}$) and aromatic moieties (1611, 1517, 1489 $cm^{-1}$), and a very intense absorption band at 1138 $cm^{-1}$ suggesting a C-O-C functional group. Its $^1H$ NMR spectrum (Table 2) showed proton signals corresponding to an oxybenzyl methine ($\delta_H$ 4.85, d, 10.1 Hz, 1H) and two methyl protons ($\delta_H$ 1.30, s, 3H; 0.95, d, 6.8 Hz, 3H), which suggested that **1** was an asymmetric tetrahydrofuran lignan.[16,17] The chemical shifts observed for aromatic protons at $\delta_H$ 7.06 (overlapped, 1H), 6.83 (d, 8.0 Hz, 1H), 7.08 (overlapped, 1H), 7.00 (s, 1H), 6.85 (d, 8.0 Hz, 1H) and 6.95 (d, 8.0 Hz, 1H), associated with the presence of intense signals corresponding to two methoxy groups ($\delta_H$ 3.91, s, 3H; $\delta_H$ 3.88, s, 3H) and a methylenedioxy moiety (5.98, s, 2H) indicated the two aromatic rings were both trisubstituted (including an

*To whom correspondence should be addressed. E-mail: xwl@mail.kib.ac.cn (W.L. Xiao); hdsun@mail.kib.ac.cn (H.D. Sun)

Springer

1,3,4-trisubstituted one). The $^{13}C$ NMR spectrum (Table 1) corroborated the assignments made for the structural determination of both aromatic rings. As expected, the two trisubstituted aromatic rings displayed the chemical shifts of six methines for aromatic carbons ($\delta_C$ 107.9, 107.9, 122.0, 110.2, 110.7, 120.2). $^1H$-$^1H$ COSY correlation of H-5 ($\delta_H$ 6.83) with H-6 ($\delta_H$ 7.08) and HMBC correlations of both H-2 ($\delta_H$ 7.06) and H-5 with C-3 and C-4 suggested that another aromatic ring was also 1,3,4-trisubstituted (Fig. 2). Furthermore, HMBC correlations from H-7′ to C-2′, C-1′, and C-6′ and from H-2 and H-6 to C-7, located the two aromatic rings to the tetrahydrofuran ring (Fig. 2). HMBC correlations from the methylenedioxy protons to C-3 and C-4 and from the methoxy protons to C-3′ and C-4′, suggested that two methoxy groups were attached at C-3′ and C-4′, while methylenedioxy moiety attached at C-3 and C-4, respectively. The proposal was confirmed by the ROESY correlations of methoxy protons ($\delta_H$ 3.91) with H-2′, of another methoxy protons ($\delta_H$ 3.88) with H-5′ (Fig. 2). Except for the presence of the above two

**1** **4**

**Figure 2.** Selected HMBC ( ⌒ ) and $^1H$-$^1H$ COSY ( ▬ ) correlations of **1** and **4**

methoxy groups, a special upfield signal belonging to another methoxy group ($\delta_H$ 3.23, $\delta_C$ 50.2) was observed in $^1H$ and $^{13}C$ NMR spectra. This upfield shifted methoxy group was deduced to be attached at C-7 by HMBC correlation observed from the methoxy signal to C-7 and the relative downfield shift of C-7 ($\delta_C$ 111.6, s). In addition, HMBC correlations (Fig. 2) from H-9, H-9′, and H-7′ to the oxygenated quaternary carbon C-8 ($\delta_C$ 82.3, s), along with the analysis of its molecular formula, indicated that C-8 of **1** was substituted by a hydroxyl group. The relative configurations of **1** were established on the basis of ROESY spectrum. The key correlations from H-9' to H-7' and H-9 revealed that they were cofacial and the correlation between H-6' and OMe-7 indicated that they were on the other side. Thus, the relative configuration of **1** was determined. H-7', H-9', and H-9 were arbitrarily defined as *β*-orientation, and accordingly, H-8' and OMe-7 were assigned as *α*-orientation (Fig. 3). Therefore, the structure of **1** was established.

Schpropinrin B (**2**), obtained as yellow oil, had the molecular formula $C_{22}H_{26}O_7$, as derived from HREIMS at *m*/*z* 402.1673 $[M]^+$, which was almost the same as that of **1**. Detailed comparison of the NMR data of **2** with those of **1** disclosed that the main structural difference between them was likely to be the locations of the substitutions in aromatic rings. HMBC correlations from the methylenedioxy protons to C-3′ and C-4′, from H-7′ to C-1′, C-2′, and C-6′, and from H-2′ and H-5′ to C-3′ and C-4′ established that the methylenedioxy group was located between C-3′ and C-4′. Furthermore, in HMBC spectrum, the correlations from H-2 and H-6 to C-7, from two methoxy groups to C-3 and C-4, and from H-2 and H-5 to C-3 and C-4, together with the ROESY correlations of

**Table 1.** $^{13}$**C NMR spectroscopic assignments of compounds 1–4**[a,b]

| position | 1 | 2 | 3 | 4 |
|---|---|---|---|---|
| 1 | 129.7 s | 130.1 s | 130.3 s | 133.2 s |
| 2 | 107.9 d | 113.3 d | 108.9 d | 110.0 d |
| 3 | 147.8 s | 150.1 s | 146.4 s | 147.5 s |
| 4 | 147.9 s | 149.6 s | 146.6 s | 148.1 s |
| 5 | 107.9 d | 111.7 d | 108.0 d | 107.7 d |
| 6 | 122.0 d | 121.8 d | 121.8 d | 121.2 d |
| 7 | 111.6 s | 112.7 s | 112.1 s | 108.0 s |
| 8 | 82.3 s | 82.6 s | 82.3 s | 81.8 s |
| 9 | 19.8 q | 19.8 q | 19.8 q | 19.8 q |
| 1′ | 133.6 s | 137.1 s | 133.0 s | 133.9 s |
| 2′ | 110.2 d | 108.1 d | 109.7 d | 110.7 d |
| 3′ | 149.1 s | 148.1 s | 147.9 s | 149.0 s |
| 4′ | 148.8 s | 148.8 s | 147.8 s | 148.7 s |
| 5′ | 110.7 d | 108.5 d | 114.0 d | 108.4 d |
| 6′ | 120.2 d | 122.0 d | 120.9 d | 119.6 d |
| 7′ | 87.6 d | 88.1 d | 87.7 d | 87.4 d |
| 8′ | 48.6 d | 50.0 d | 48.6 d | 48.6 d |
| 9′ | 8.3 q | 8.6 q | 8.3 q | 8.3 q |
| 3-OCH$_2$O- | 101.2 t | 56.0 q (3′-OCH$_3$) | 101.2 t | 101.2 t |
| 4-OCH$_2$O- | | 55.9 q (4′-OCH$_3$) | | |
| 3′-OCH$_3$ | 55.7 q | 102.0 t (-OCH$_2$O-) | 55.8 q | 55.8 q |
| 4′-OCH$_3$ | 55.9 q | | | 55.9 q |
| 7-OCH$_3$ | 50.2 q | 50.2 q | 50.2 q | |

[a]Recorded in $CD_3Cl$; [b]Recorded at 100 MHz.

one methoxy group ($\delta_H$ 3.80) with H-2, of another methoxy group ($\delta_H$ 3.79) with H-5, confirmed that two methoxy groups were located at C-3 and C-4, respectively. In the ROESY experiment, H-9′ showed strong correlations with H-9 and H-7′, and H-7′ with H-2′, indicating *β*-orientation of the H-9, H-9′, and H-7′. OMe-7 ($\delta_H$ 3.16, s, 3H) showed correlations with H-2 and H-6′, indicating its *α*-orientation.

**1** **4**

**Figure 3.** Selected ROESY ( ⟷ ) correlations of **1** and **4**

Schpropinrin C (**3**) was obtained as yellow amorphous powder. Its molecular formula, $C_{21}H_{24}O_7$, was determined by the molecular ion peak $[M]^+$ in the positive HREIMS at *m*/*z* 388.1514 (calcd 388.1522), indicating 10 degrees of unsaturation. Detailed comparison of $^1H$ and $^{13}C$ NMR data of **3** with those of **1** showed they are structurally similar. The major difference was the disappearance of a methoxy signal ($\delta_C$ 55.8, q) in **3**. HMBC correlations from H-7′ to C-2′, and C-6′, from H-2′ and H-5′ to C-3′ and C-4′, and from the methoxy protons ($\delta_H$ 3.92, s, 3H) to C-4′, together with the correlations of the methoxy protons with H-2′ in the ROESY spectrum, determined that the hydroxyl group and the methoxy group

**Table 2. $^1$H NMR spectroscopic assignments of compounds 1–4[a,b]**

| position | 1 | 2 | 3 | 4 |
|---|---|---|---|---|
| 2 | 7.06[c] | 7.07 (d, 7.0) | 7.05[c] | 7.08 (s) |
| 5 | 6.83 (d, 8.0) | 6.92[c] | 6.84 (d, 8.0) | 6.82[c] |
| 6 | 7.08[c] | 7.04 (d, 7.0) | 7.08[c] | 7.08[c] |
| 9 | 1.30 (s) | 1.31 (s) | 1.30 (s) | 1.29 (s) |
| 2′ | 7.00 (s) | 6.95 (s) | 6.98 (s) | |
| 5′ | 6.85 (d, 8.0) | 6.83 (d, 7.0) | 6.91[c] | 6.84[c] |
| 6′ | 6.95 (d, 8.0) | 6.91 (d, 7.0) | 6.91[c] | 6.94 (d, 8.2) |
| 7′ | 4.85 (d, 10.1) | 4.83 (d, 10.2) | 4.85 (d, 10.2) | 4.84 (d, 10.2) |
| 8′ | 2.45 (m) | 2.35 (m) | 2.45 (m) | 2.45 (m) |
| 9′ | 0.95 (d, 6.8) | 0.90 (d, 6.8) | 0.95 (d, 6.8) | 0.94 (d, 6.8) |
| $OCH_2O$-3,4 | 5.98 (s) | 6.00 (s) ($OCH_2O$-3′,4′) | 5.98 (s) | 5.96 (s) |
| 7-$OCH_3$ | 3.23 (s) | 3.16 (s) | 3.22 (s) | |
| 3′-$OCH_3$ | 3.91 (s) | 3.80 (s) (3-$OCH_3$) | 3.92 (s) | 3.90 (s) |
| 4′ -$OCH_3$ | 3.88 (s) | 3.79 (s) (4-$OCH_3$) | | 3.87 (s) |

[a]Recorded in $CD_3Cl$; [b]Recorded at 400 MHz; [c]overlapped were located at C-4′ and C-3′, respectively. The relative configurations were established to be the same as compound **1** by analysis of the ROESY experiment.

Schpropinrin D (**4**), yellow oil, had the formula $C_{21}H_{24}O_7$ for HREIMS *m*/*z* 388.1520 $[M]^+$, suggesting 10 degrees of unsaturation. Comparison of $^1$H and $^{13}$C NMR data of **4** with those of **1** suggested that **4** was structurally related to **1**. The difference between **1** and **4** could be explained by the replacement of a methoxy group in **1** by a OH group in **4**, which was further confirmed by the conducting of sets of 2D NMR experiments ($^1$H-$^1$H COSY, HSQC, HMBC, and ROESY spectra) and the relative upfield shift of C-7 ($\delta_C$ 108.0, s) in **4** (Figs. 2 and 3).

On the basis of the relative configuration was established, the quantum chemical calculation of electronic circular dichroism (ECD) spectrum was carried out for schpropinrin D (**4**) using the time dependent DFT (TDDFT) method with the B3LYP-SCRF/6-31+G(d,p)//B3LYP/6-31G(d) level.[18,19] The calculated ECD spectrum for the 7*S*, 8*R*, 7'*R*, 8'*S* enantiomer correspond with the experimental ECD curve (Fig. 4). In addition, the experimental ECD spectra of compounds **1**–**3** were nearly identical with that of **4**, suggesting that the stereochemistry of compounds **1**–**3** were also with 7*S*, 8*R*, 7'*R*, 8'*S* configurations.

**Figure 4.** Calculated and experimental ECD spectra of **4**

Compounds **1**–**9** at a concentration of 25 $\mu$M were evaluated for their ability to inhibit HIV-1 IN DNA binding activity in vitro using the method previously used.[20] All of the compounds were inactive at the concentration tested.

## Experimental Section

**General Experimental Procedures.** Optical rotations were measured with a Horiba SEPA-300 polarimeter. UV spectrum were obtained using a Shimadzu UV-2401A spectrophotometer. A Tenor 27 spectrophotometer was used for scanning IR spectroscopy with KBr pellets. 1D and 2D NMR spectra were recorded on Bruker AM-400, DRX-500 and Avance III-600 spectrometers. Unless otherwise specified, chemical shifts ($\delta$) were expressed in ppm with reference to the solvent signals. Mass spectra were performed on a VG Autospec-3000 spectrometer at 70 eV. Column chromatography was performed using silica gel (200–300 mesh, Qing-dao Marine Chemical, Inc., Qingdao, China). Semi-preparative HPLC was performed on an Agilent 1100 liquid chromatography with a Zorbax SB-$C_{18}$, 9.4 mm × 25 cm column. Preparative HPLC was performed on a Shimadzu LC-8A preparative liquid chromatography with a Shimadzu PRC-ODS (K) column. Fractions were monitored by TLC and spots were visualized by heating the silica gel plates sprayed with 10% $H_2SO_4$ in EtOH.

**Plant Material.** The leaves and stems of *S. propinqua* var. *sinensis* were collected in Shennongjia, Hubei Province, China, in September 2010. The specimen was identified by Prof. Heng Li and a voucher specimen (No. KIB 2010-09-15) has been deposited at the State Key Laboratory of Phytochemistry and Plant Resources in West China, Kunming Institute of Botany, Chinese Academy of Sciences.

**Extraction and Isolation.** The plant material of *S. propinqua* var. *sinensis* (15 kg) was ground and exhaustively extracted with $Me_2CO$-$H_2O$ (V/V = 7:3) at room temperature. The solvent was evaporated *in vacuo*, and the crude extract was dissolved in $H_2O$ and partitioned with EtOAc. The EtOAc portion (480 g) was chromatographed on a silica gel column being eluted with $CHCl_3$-$Me_2CO$ (1:0, 9:1, 8:2, 2:1, 1:1, and 0:1) to afford fractions I–VII. Fraction II (32 g) was applied to RP-18, eluted with a MeOH-$H_2O$ (40%–100%) gradient system, to afford five fractions. Fraction II-4 (6.3 g) were further repeatedly chromatographed on silica gel, Sephadex LH-20, and finally by semi-preparative HPLC to yield **1** (9

mg), **2** (6 mg), **3** (10 mg), **5** (20 mg), **6** (11 mg), **7** (4 mg) and **8** (14 mg). Compounds **4** (7 mg) and **9** (3 mg) were purified from Fraction III (14 g) through successively subjected to silica gel, RP-18 and Sephadex LH-20.

**Schpropinrin A (1):** yellow oil; $[\alpha]_D^{24}$ + 66.0 (*c* 0.14, MeOH); UV (MeOH) $\lambda_{max}$ (log $\varepsilon$) 282 (3.11), 231 (3.43), 204 (3.96) nm; CD (MeOH) $\lambda_{max}$ ($\Delta\varepsilon$) 286 (+ 0.97), 234 (+ 2.87), 205 (+ 20.40) nm; IR (KBr) $\nu_{max}$ 3443, 2961, 2934, 2879, 1611, 1517, 1489, 1463, 1439, 1250, 1138 cm$^{-1}$; $^1$H and $^{13}$C NMR data, see Tables 1 and 2; HREIMS *m/z* 402.1688 [M]$^+$ (calcd for $C_{22}H_{26}O_7$, 402.1679).

**Schpropinrin B (2):** yellow oil; $[\alpha]_D^{24}$ + 76.8 (*c* 0.20, MeOH); UV (MeOH) $\lambda_{max}$ (log $\varepsilon$) 282 (3.11), 231 (3.41), 205 (3.98) nm; CD (MeOH) $\lambda_{max}$ ($\Delta\varepsilon$) 286 (+ 0.74), 234 (+ 2.23), 205 (+ 15.25) nm; IR (KBr) $\nu_{max}$ 3444, 2962, 2935, 2879, 1608, 1515, 1490, 1462, 1444, 1250, 1138 cm$^{-1}$; $^1$H and $^{13}$C NMR data, see Tables 1 and 2; HREIMS *m/z* 402.1673 [M]$^+$ (calcd for $C_{22}H_{26}O_7$, 402.1679).

**Schpropinrin C (3):** yellow amorphous powder; $[\alpha]_D^{25}$ + 41.3 (*c* 0.30, MeOH); UV (MeOH) $\lambda_{max}$ (log $\varepsilon$) 283 (2.99), 229 (3.28), 202 (3.90) nm; CD (MeOH) $\lambda_{max}$ nm ($\Delta\varepsilon$) 282 (+ 0.62), 232 (+ 2.04), 205 (+ 14.16); IR (KBr) $\nu_{max}$ 3442, 2963, 2935, 1614, 1516, 1489, 1463, 1437, 1250, 1135 cm$^{-1}$; $^1$H and $^{13}$C NMR data, see Tables 1 and 2; HREIMS *m/z* 388.1514 [M]$^+$ (calcd for $C_{21}H_{24}O_7$, 388.1522).

**Schpropinrin D (4):** yellow oil; $[\alpha]_D^{24}$ + 1.4 (*c* 0.23, MeOH); UV (MeOH) $\lambda_{max}$ (log $\varepsilon$) 282 (3.17), 231 (3.45), 204 (3.90) nm; CD (MeOH) $\lambda_{max}$ ($\Delta\varepsilon$) 282 (+ 0.65), 233 (+ 1.92), 205 (+ 13.48) nm; IR (KBr) $\nu_{max}$ 3449, 2964, 2936, 1599, 1511, 1489, 1463, 1443, 1249, 1127 cm$^{-1}$; $^1$H and $^{13}$C NMR data, see Tables 1 and 2; HREIMS *m/z* 388.1520 [M]$^+$ (calcd for $C_{21}H_{24}O_7$, 388.1522).

**ECD Calculation.** The theoretical calculations of compound **4** were performed using Gaussian 09.[21] The conformers were optimized at the B3LYP/6-31G (d) level. Room temperature equilibrium populations were calculated according to Boltzmann distribution law. The theoretical calculation of ECD was performed using time dependent Density Functional Theory (TDDFT)[18,19] at the B3LYP/6-31G+(d, p) level in MeOH with PCM model and in the gas phase, respectively. The ECD spectra of compound **4** were obtained by weighing the Boltzmann distribution rate of each geometric conformation.

**ECD Simulation.** The ECD spectra are simulated by overlapping Gaussian functions for each transition according to:

$$\Delta\varepsilon(E) = \frac{1}{2.297\times10^{-39}} \times \frac{1}{\sqrt{2\pi}\sigma}\sum_i^A \Delta E_i R_i e^{-[(E-E_i)/(2\sigma)]^2}$$

The $\sigma$ represented the width of the band at 1/*e* height, and $\Delta E_i$ and $R_i$ are the excitation energies and rotational strengths for transition *i*, respectively. $\sigma$ = 0.20 eV and $R^{velocity}$ have been used in this work.

**Microplate-Based Assay for HIV-1 IN-DNA Binding:** IN-DNA binding assays were performed using purified recombinant integrase protein as previously described.[20] Briefly, IN (500 nM) in PBS (pH 7.4) was loaded into Nunc FluoroNunc microplate wells (200 $\mu$L per well) and the plates were incubated at 4 °C for overnight. Unbound proteins were removed by three washes in 200 $\mu$L PBS per well (pH 7.4). The plates were then blocked with 200 $\mu$L PBS/well containing 2% BSA (wt/vol) for 2 h and washed three times in PBS. Drugs were prepared as a 5X work solution at 125 $\mu$M in binding buffer (20 mM MOPS [pH 7.2], 20 mM NaCl, 5 mM DTT, 7.5 mM $MgCl_2$) and 20 $\mu$L each drug was added to each well (a final concentration of 25 $\mu$M). Then, 80 $\mu$L of a 5'-rhodamine-labeled LTR duplex (20 nM) in binding buffer were added to the wells (total vol. of 100 $\mu$L per well). Wells containing 25 $\mu$L of binding buffer, but without drug, were used as positive controls (no drug). The plates were incubated at room temperature for 1 h. After removal of the reaction mixtures by rapid inversion, the plates were then washed three times in PBS. Finally, 100 $\mu$L PBS was added to each well, and fluorescence was measured in a FLUOstar Optima plate reader (BMG Labtech) at excitation and emission wavelengths of 544 and 590 nm, respectively. All measurements were performed in triplicate, and experiments were repeated at least three times. The percentages of inhibition of IN DNA binding activity by each drug were calculated by comparing activities relative to the positive control without drug (set as 100%).

**Electronic Supplementary Material**

Supplementary material is available in the online version of this article at http://dx.doi.org/ 10.1007/s13659-013-0017-8 and is accessible for authorized users.

**Acknowledgments**

This project was supported financially by the NSFC (No. 20802082 and 30830115), the projects from the Chinese Academy of Sciences (KSCX2-EW-Q-10 and KSCX1-YW-R-24), the Major State Basic Research Development Program of China (No. 2009CB522303 and 2009CB940900), the Natural Science Foundation of Yunnan Province (No. 2012FB178), and a part of this work was financially supported by the Canadian Institutes for Health Research.

**Open Access** This article is distributed under the terms of the Creative Commons Attribution License which permits any use, distribution, and reproduction in any medium, provided the original author(s) and source are credited.

## Electronic supplementary material

Supplementary material, approximately 4.15 MB.
