# Supplementary material for: Four new lignans from the leaves and stems of Schisandra propinqua var. sinensis
Source: Nat Prod Bioprospect. 2013 Apr 3;3(2):56–60. doi: 10.1007/s13659-013-0017-8 (PMC4131657; doi:10.1007/s13659-013-0017-8)

Electronic Supplementary Material

Four new lignans from the leaves and stems of *Schisandra propinqua* var. *sinensis*

Shan-Zhai SHANG,[a,c] Ying-Shan HAN,[b] Yi-Ming SHI,[a,c] Xue DU,[a] Cheng-Qin LIANG,[a,c] Mark A. WAINBERG,[b] Zhong-Hua GAO,[a,c] Wei-Lie XIAO,[a,*] and Han-Dong SUN[a,*]

[a]State Key Laboratory of Phytochemistry and Plant Resources in West China, Kunming Institute of Botany, Chinese Academy of Sciences, Kunming 650201, China

[b]McGill University AIDS Centre, Lady Davis for Medical Research, Jewish General Hospital, Montreal, Quebec, Canada

[c]University of Chinese Academy of Sciences, Beijing 100049, China

Received 26 February 2013; Accepted 27 March 2013

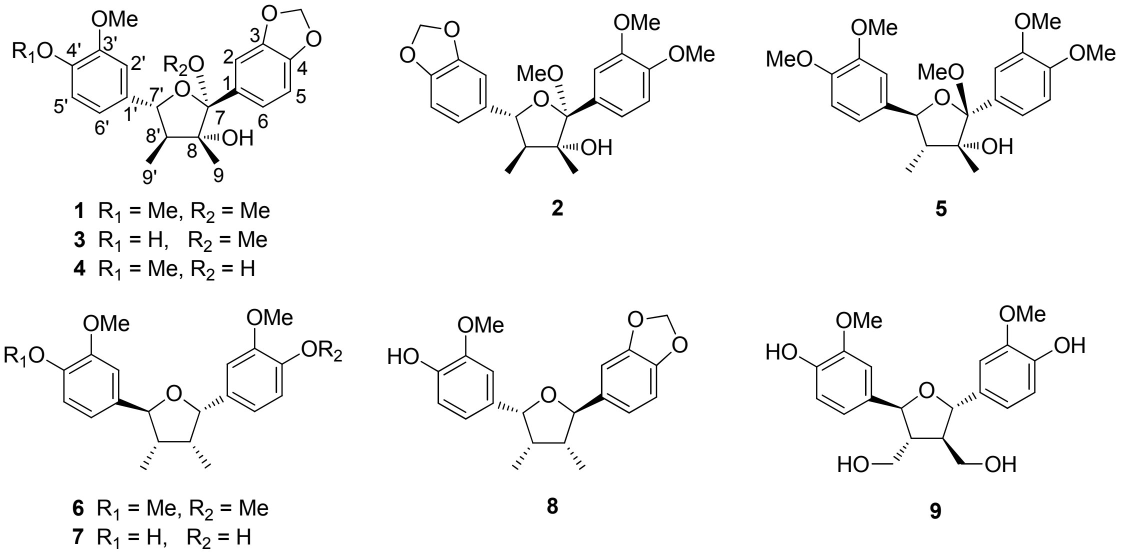

Structures of compounds **1**–**9**

*To whom correspondence should be addressed. E-mail: xwl@mail.kib.ac.cn (W.L. Xiao); hdsun@mail.kib.ac.cn (H.D. Sun)

Contents of Electronic Supplementary Material

No.	Contents:	Pages:
1.	**Figures S1-S8**. HREI MS, ^{1}H NMR, ^{13}C NMR, HSQC, ^{1}H-^{1}H COSY, HMBC, ROESY and CD spectra of **1**	3-10
2.	**Figures S9-S16**. HREI MS, ^{1}H NMR, ^{13}C NMR, HSQC, ^{1}H-^{1}H COSY, HMBC, ROESY and CD spectra of **2**	11-18
3.	**Figures S17-S20**. HREI MS, ^{1}H NMR, ^{13}C NMR and CD spectra of **3**	19-22
4.	**Figures S21-S24**. HREI MS, ^{1}H NMR, ^{13}C NMR and CD spectra of **4**	23-26

Figures S1 HRESI MS spectrum of **1**

Elemental Composition Report — **Page 1**

Single Mass Analysis
Tolerance = 10.0 PPM / DBE: min = -10.0, max = 120.0
Selected filters: None

Monoisotopic Mass, Odd and Even Electron Ions
23 formula(e) evaluated with 1 results within limits (up to 51 closest results for each mass)
Elements Used:
C: 0-200 H: 0-400 O: 5-8

sww66 — KIB — Autospec Premier
09:36:52 02-Jul-2012 — M120702EA-02AFAMM 29 (2.662) — P776
Voltage EI+ — 402.1688 — 17.3

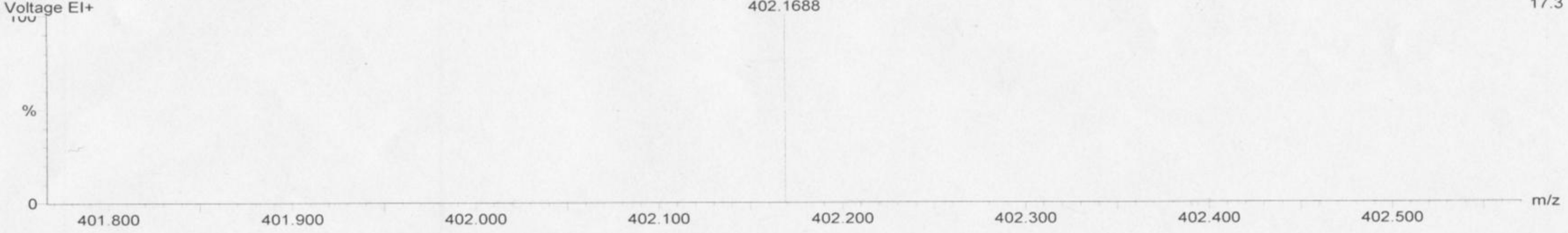

Minimum:				-10.0		
Maximum:		100.0	10.0	120.0		
Mass	Calc. Mass	mDa	PPM	DBE	i-FIT	Formula
402.1688	402.1679	0.9	2.2	10.0	5546028.0	C22 H26 O7

Figures S2 ^{1}H NMR spectrum of **1**

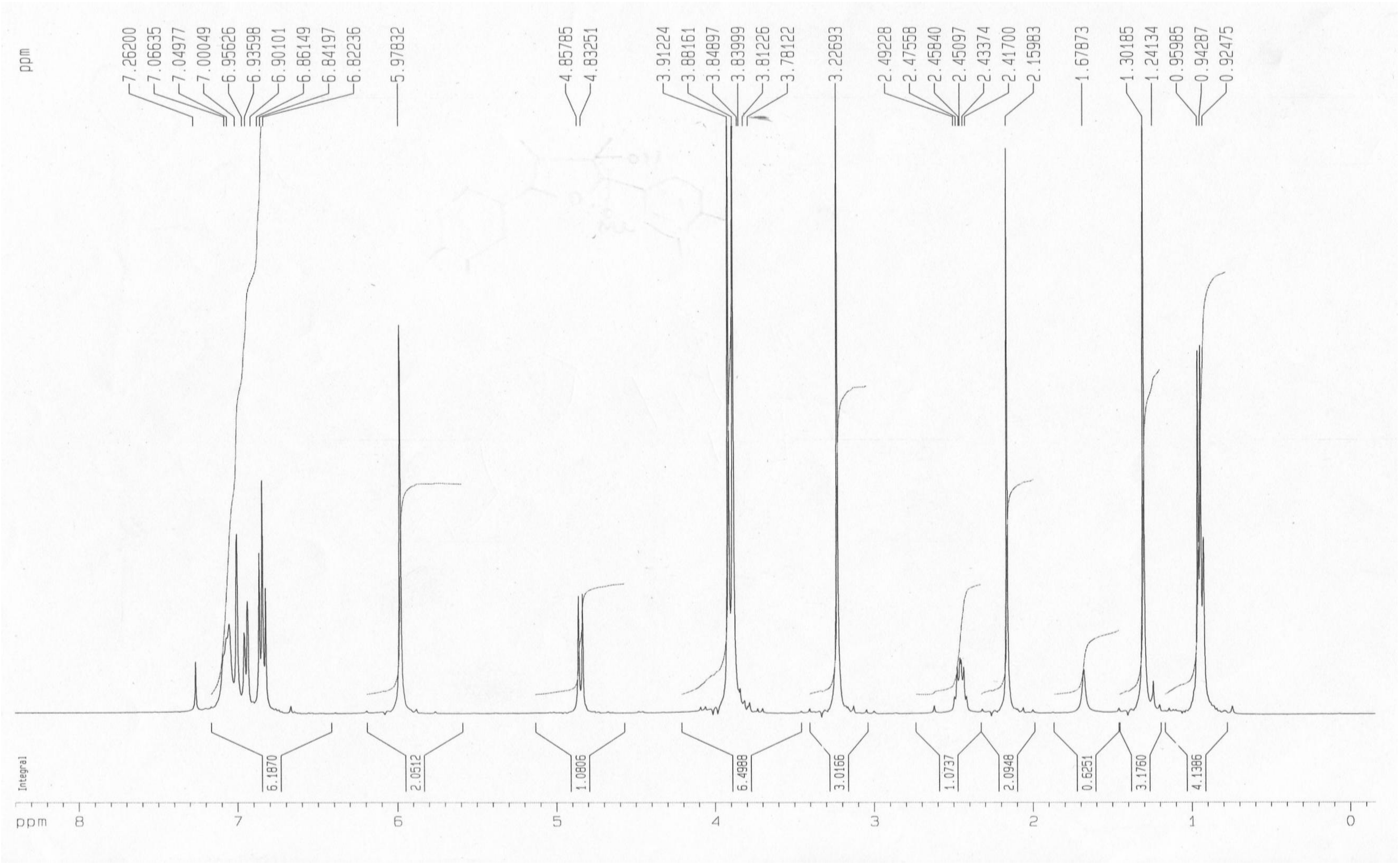

Figure S3. ^{13}C NMR spectrum of **1**

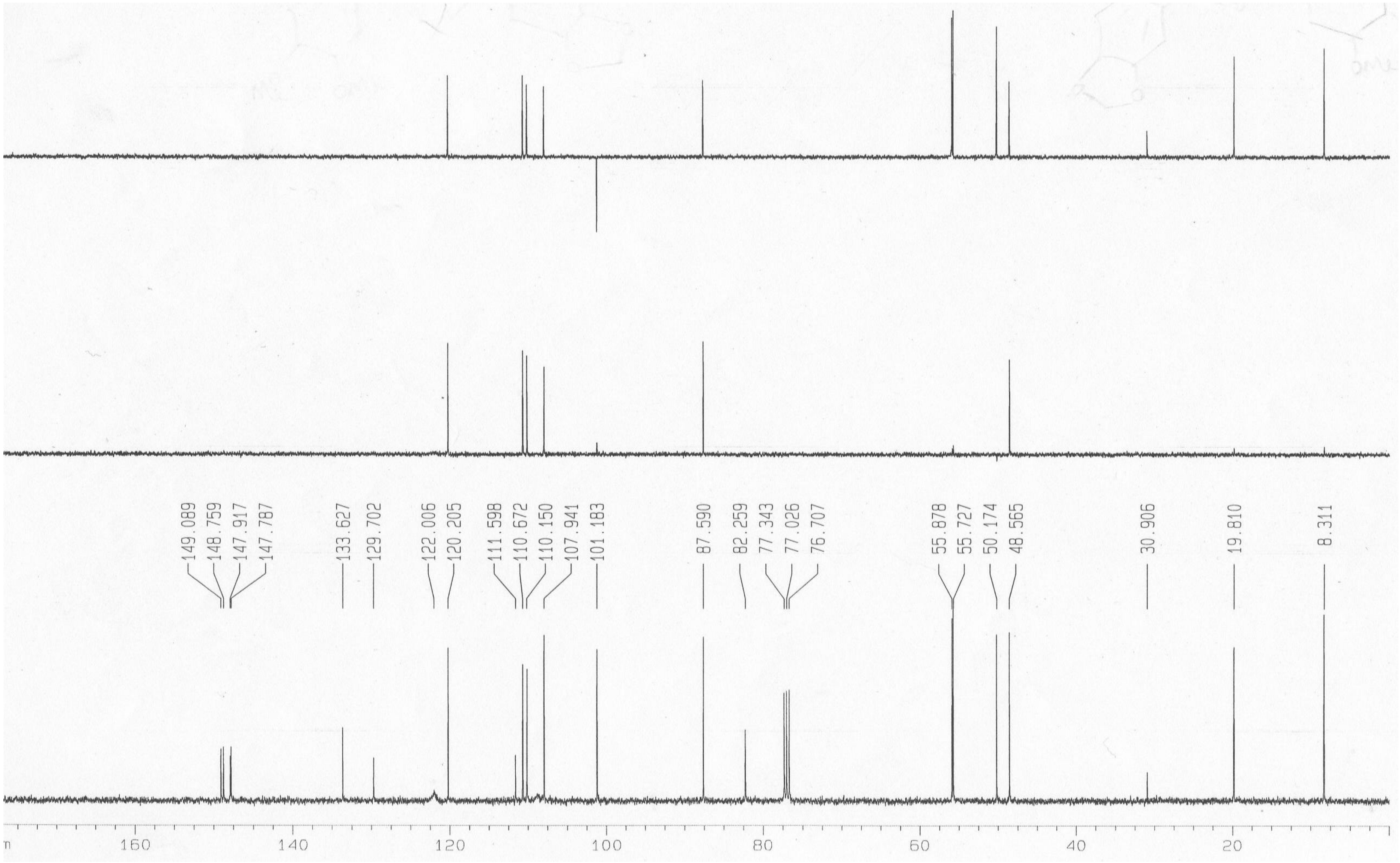

Figure S4. HSQC spectrum of **1**

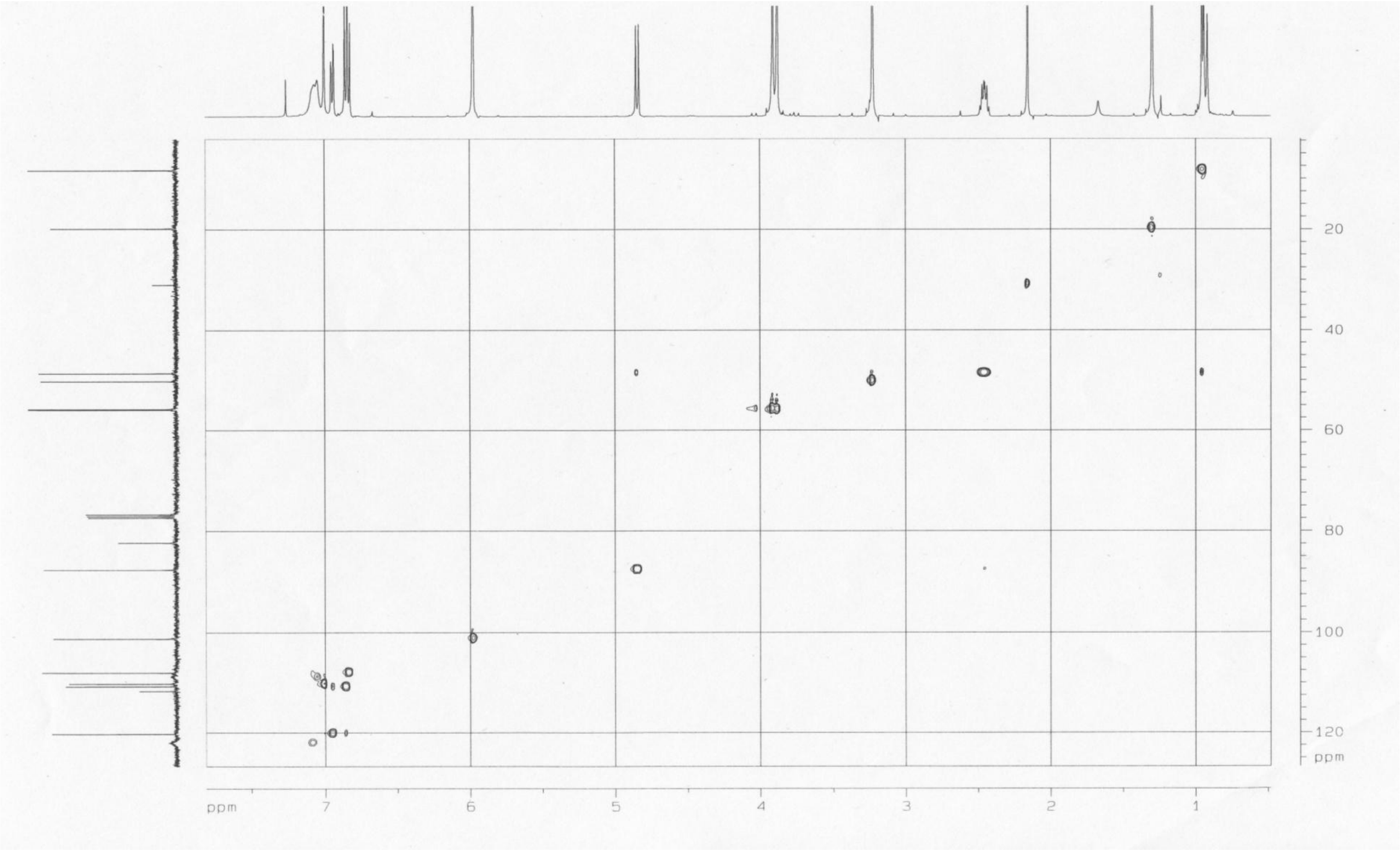

Figure S5 ^{1}H-^{1}H COSY spectrum of **1**

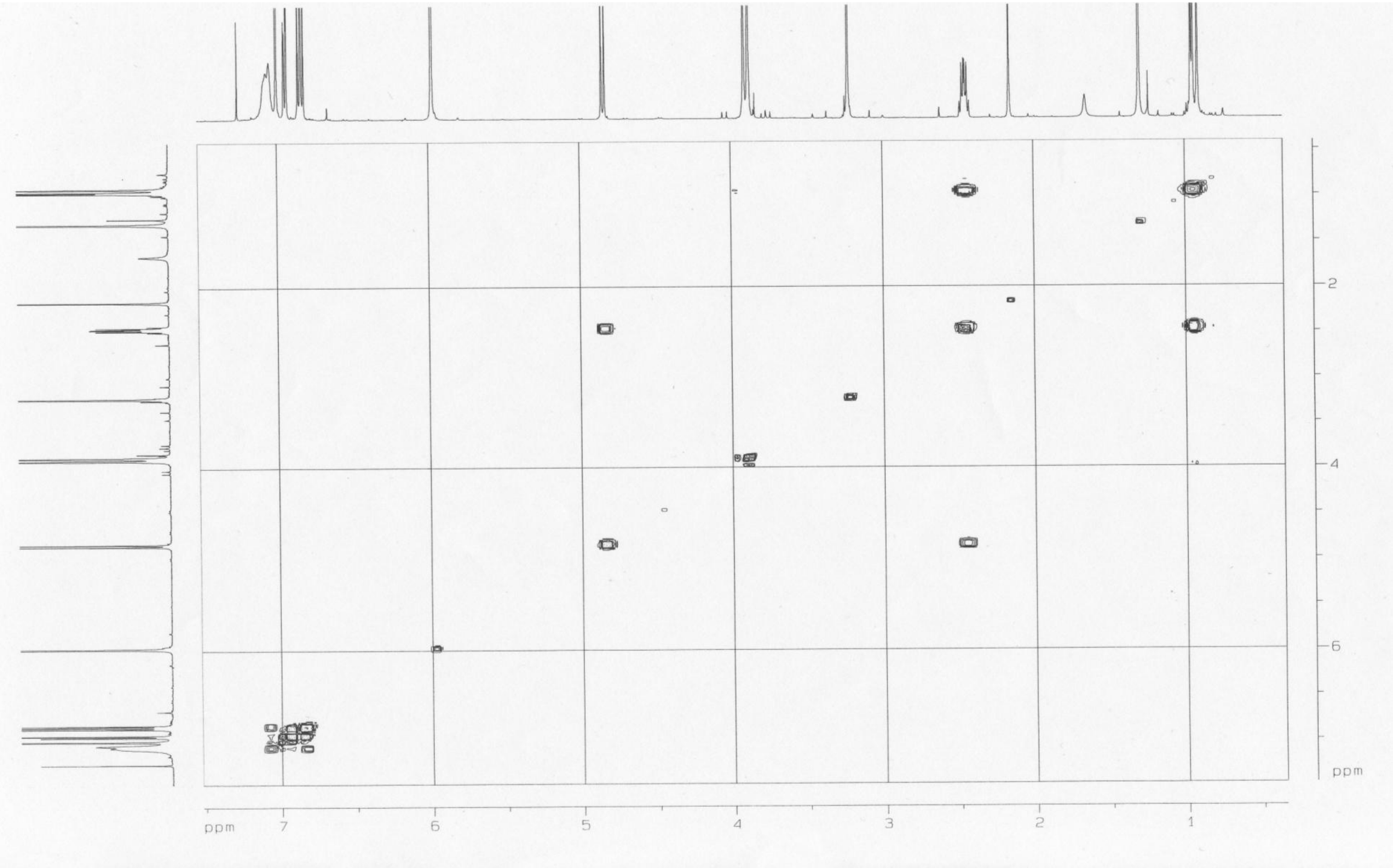

Figure S6 HMBC spectrum of **1**

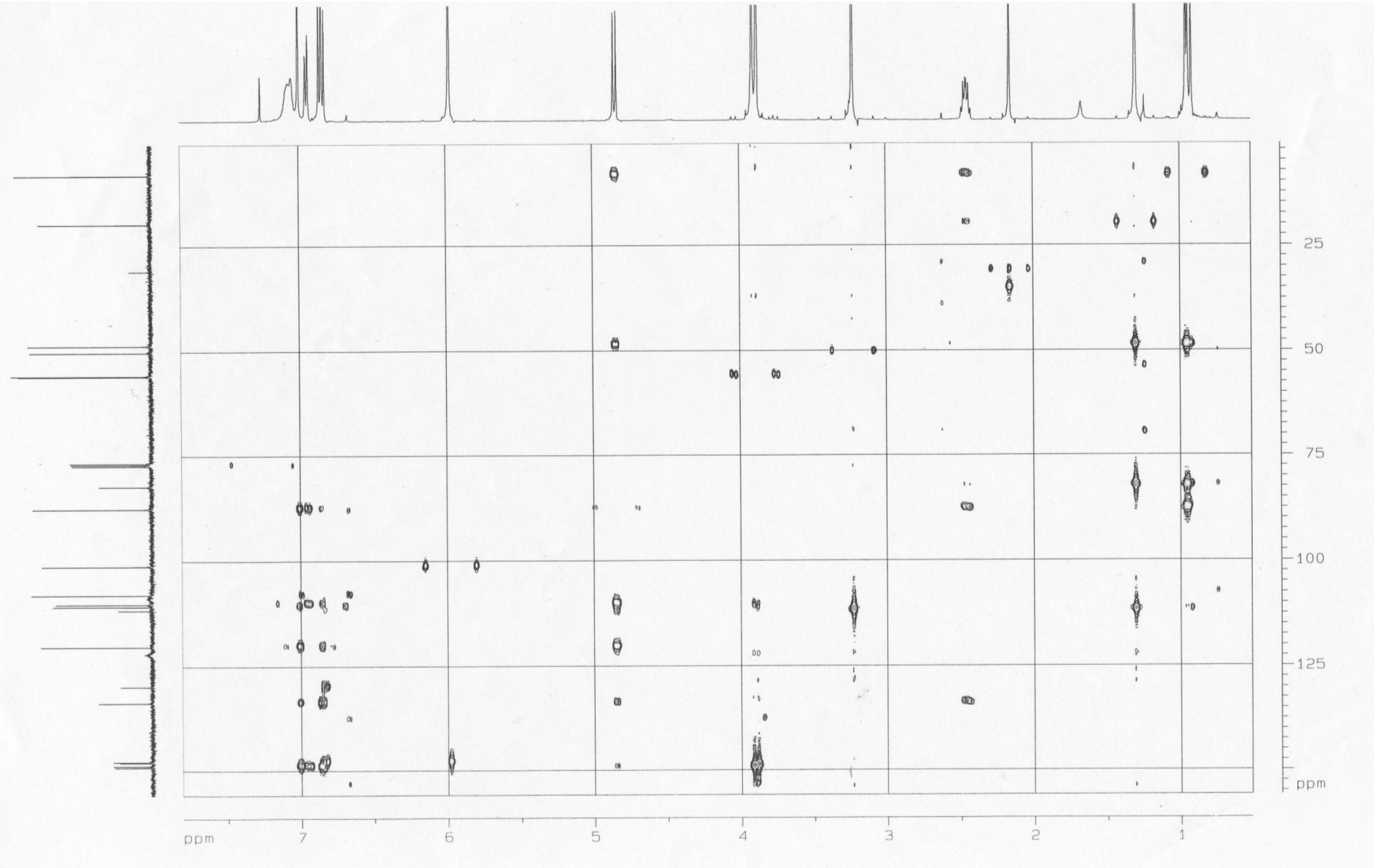

Figure S7 ROESY spectrum of **1**

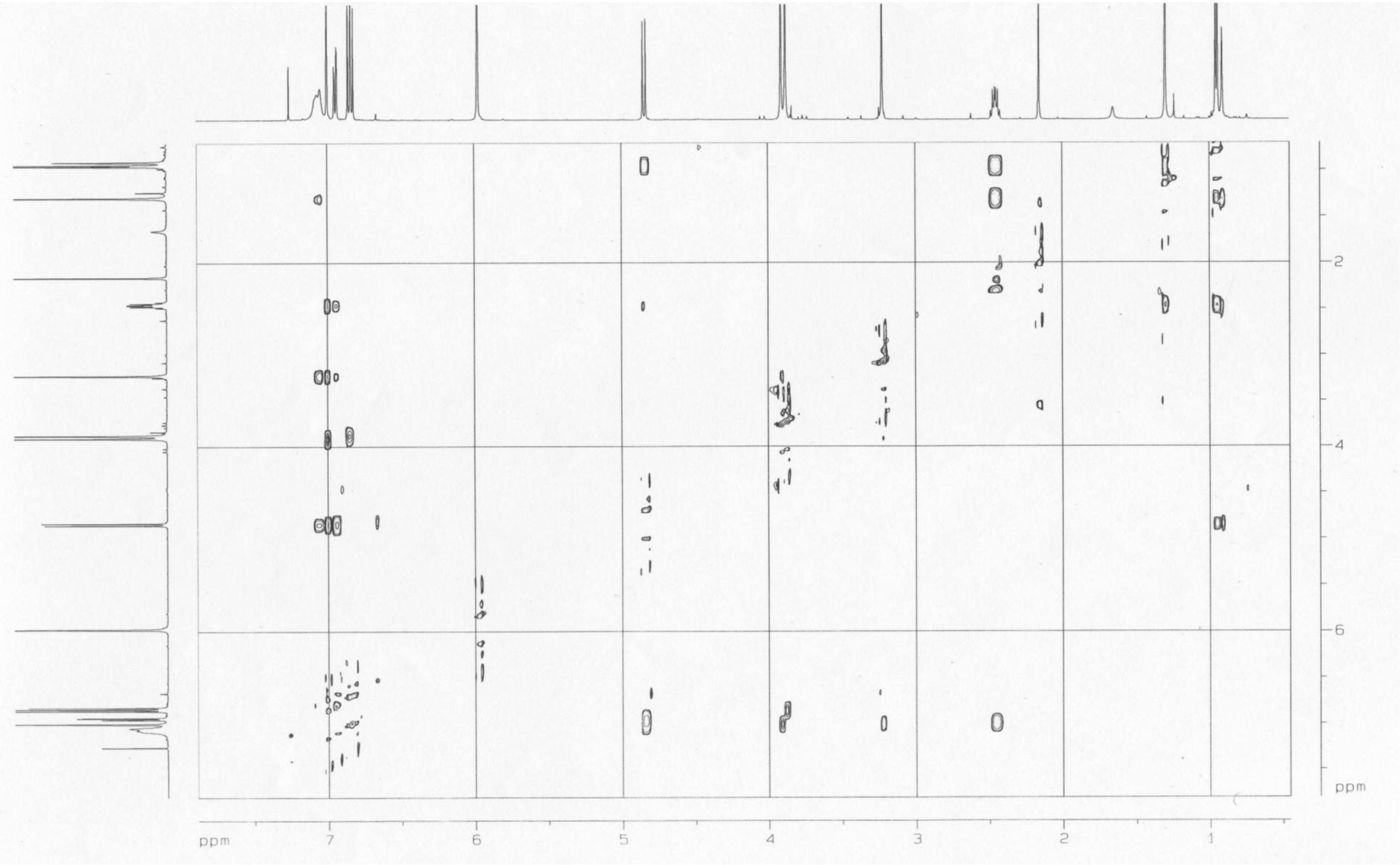

Figures S8 CD spectrum of **1**

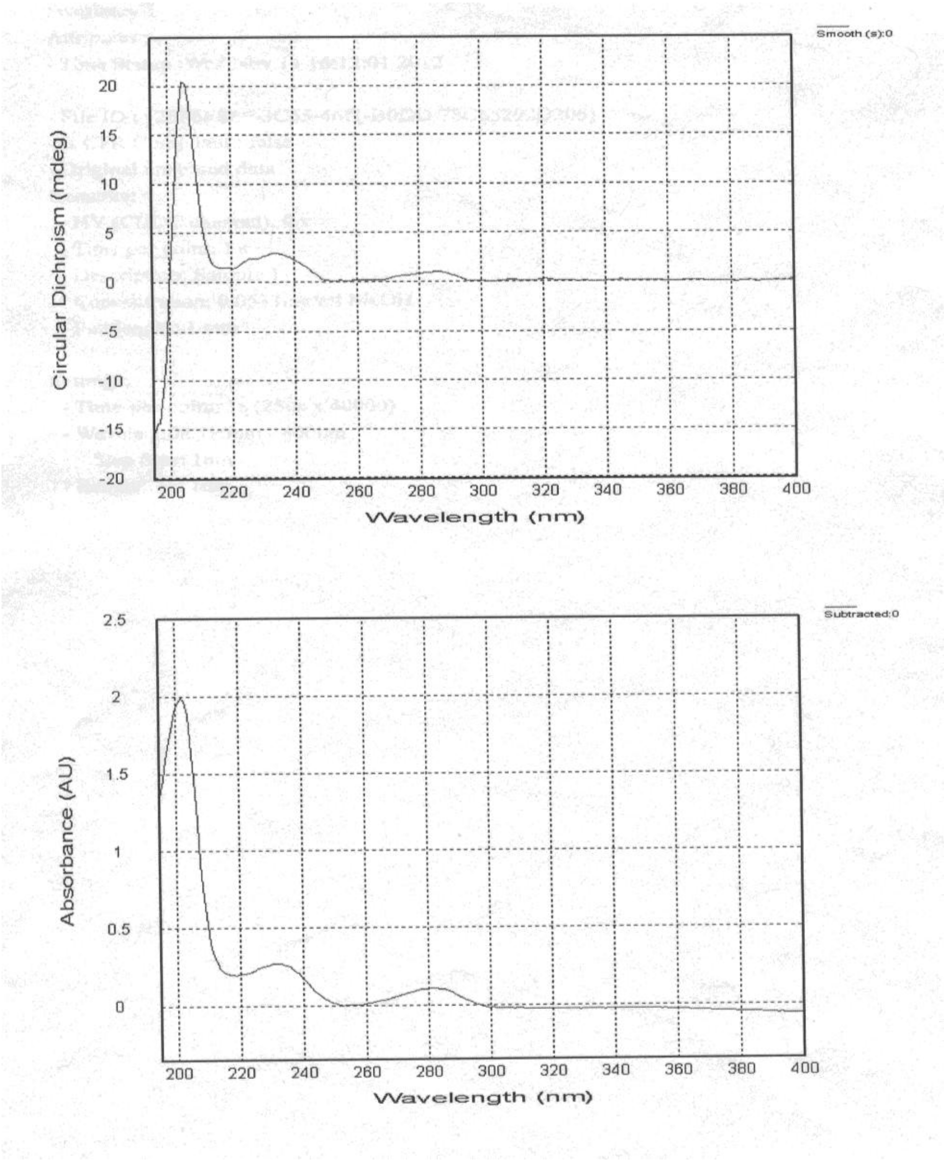

Figure S9. HREI spectrum of **2**

Elemental Composition Report

Page 1

Single Mass Analysis
Tolerance = 10.0 PPM / DBE: min = -10.0, max = 120.0
Selected filters: None

Monoisotopic Mass, Odd and Even Electron Ions
23 formula(e) evaluated with 1 results within limits (up to 51 closest results for each mass)
Elements Used:
C: 0-200 H: 0-400 O: 5-8

sww62a
09:42:23 02-Jul-2012
Voltage EI+

KIB
M120702EA-03AFAMM 16 (1.469)

Autospec Premier
P776
60

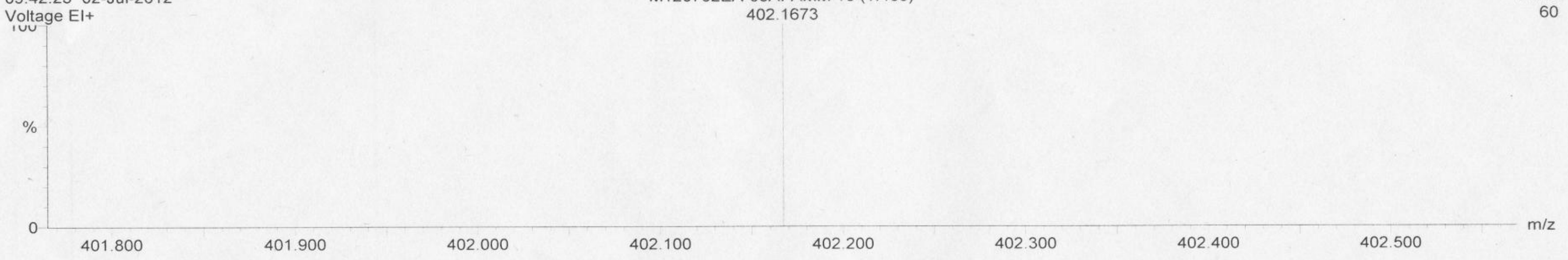

Minimum:				-10.0		
Maximum:		100.0	10.0	120.0		
Mass	Calc. Mass	mDa	PPM	DBE	i-FIT	Formula
402.1673	402.1679	-0.6	-1.5	10.0	5546045.5	C22 H26 O7

Figure S10. ^{1}H NMR spectrum of **2**

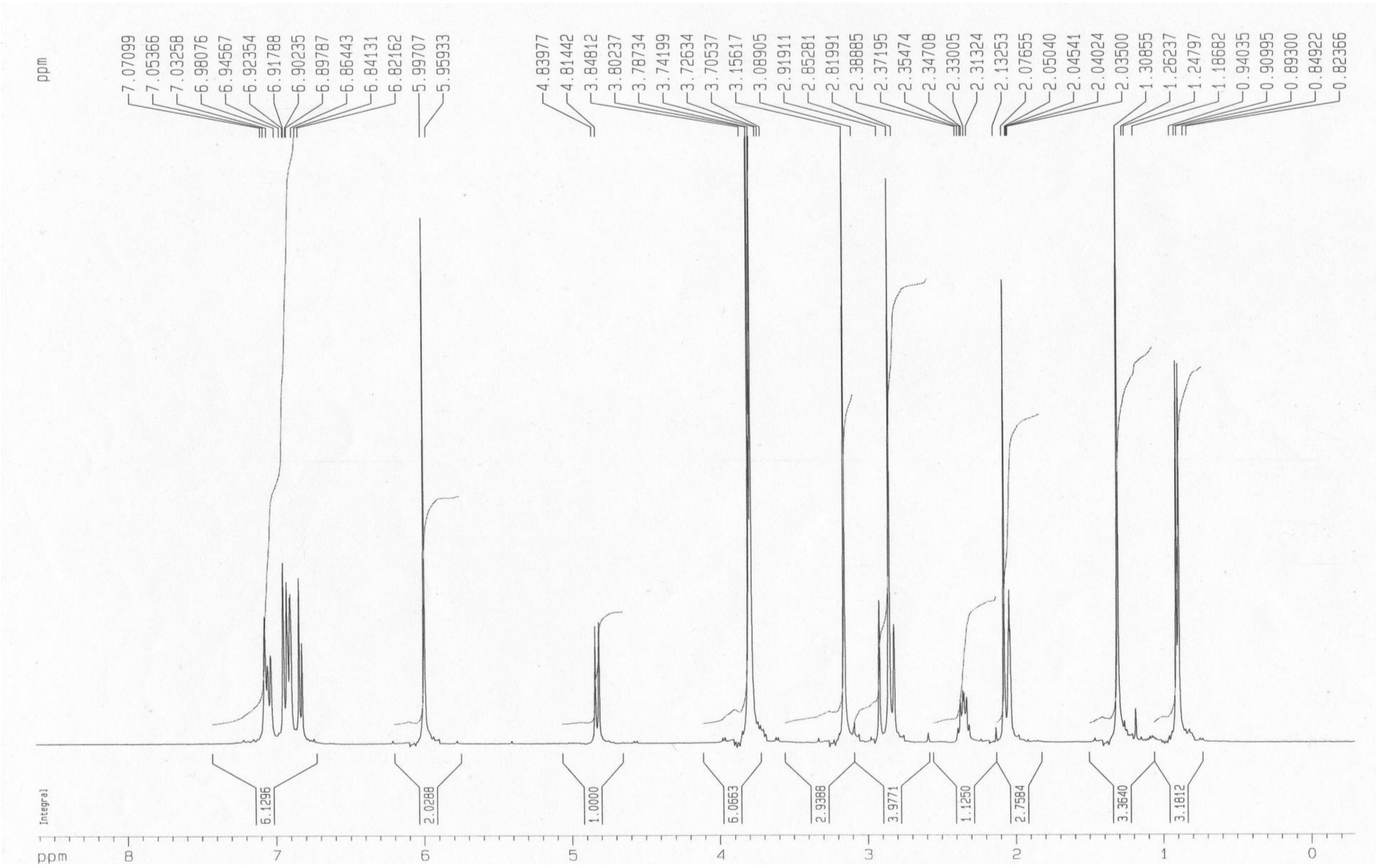

Figure S11. ^{13}C NMR spectrum of **2**

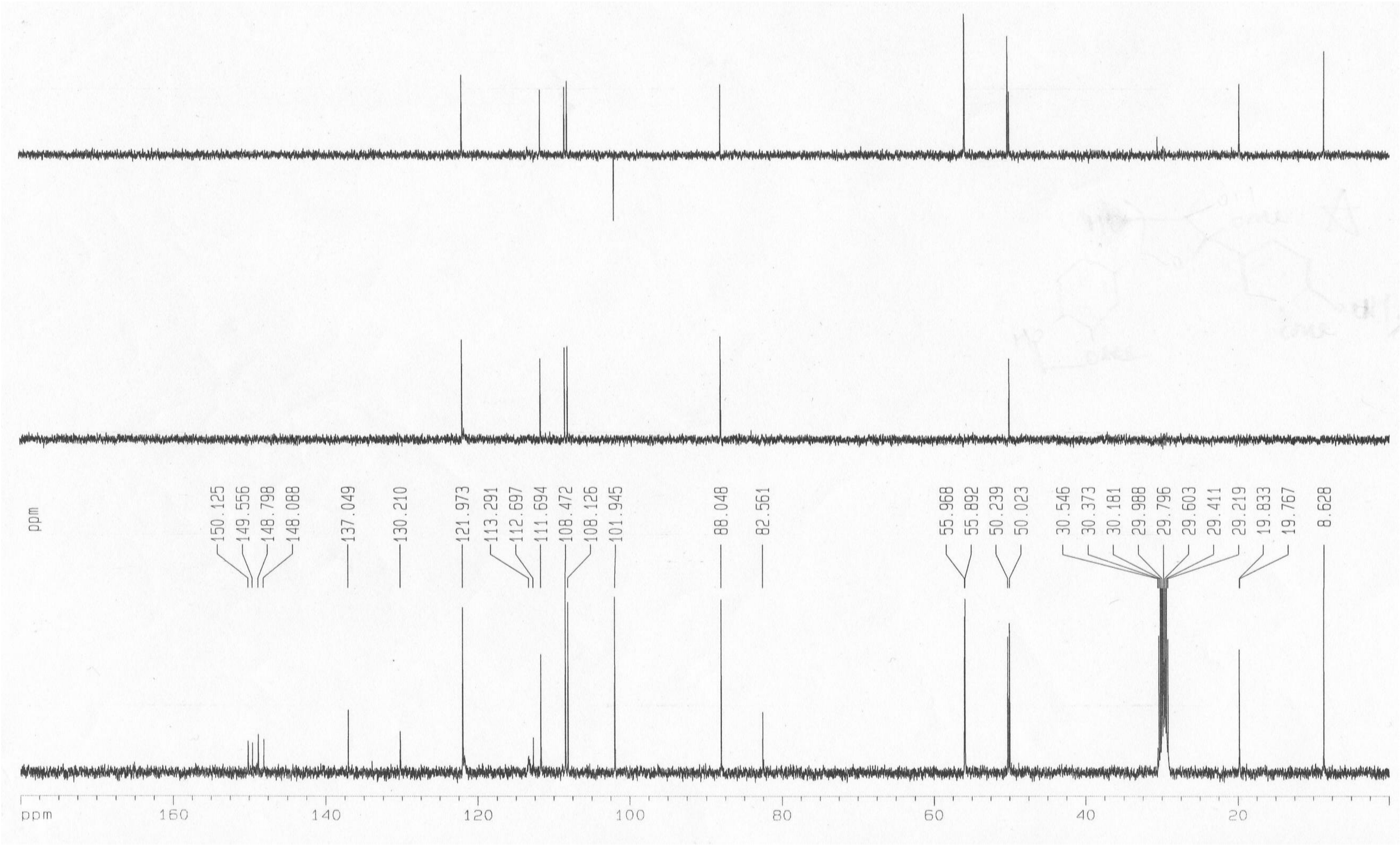

Figure S12. HSQC spectrum of **2**

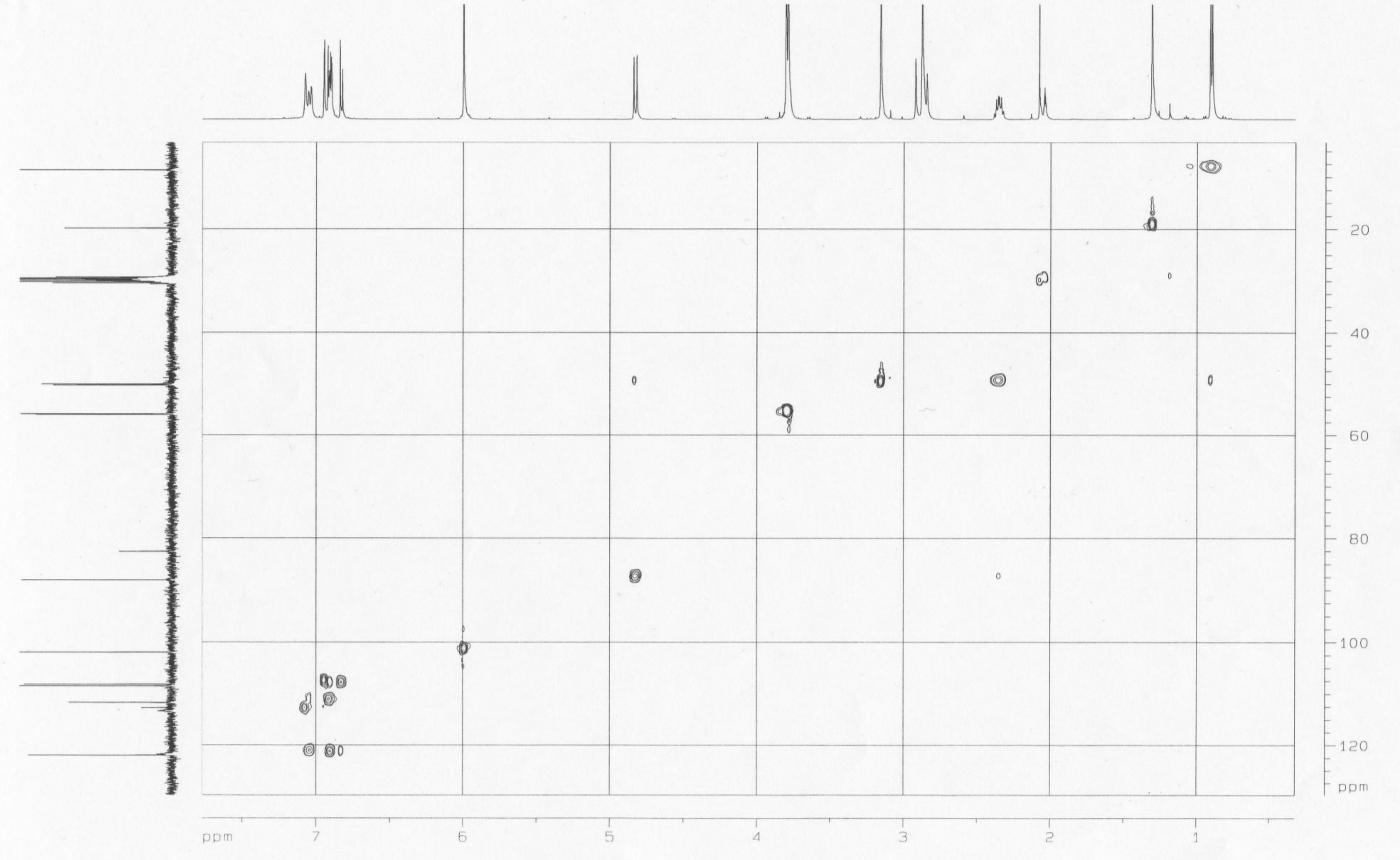

Figure S13. ^1H-^1H COSY spectrum of **2**

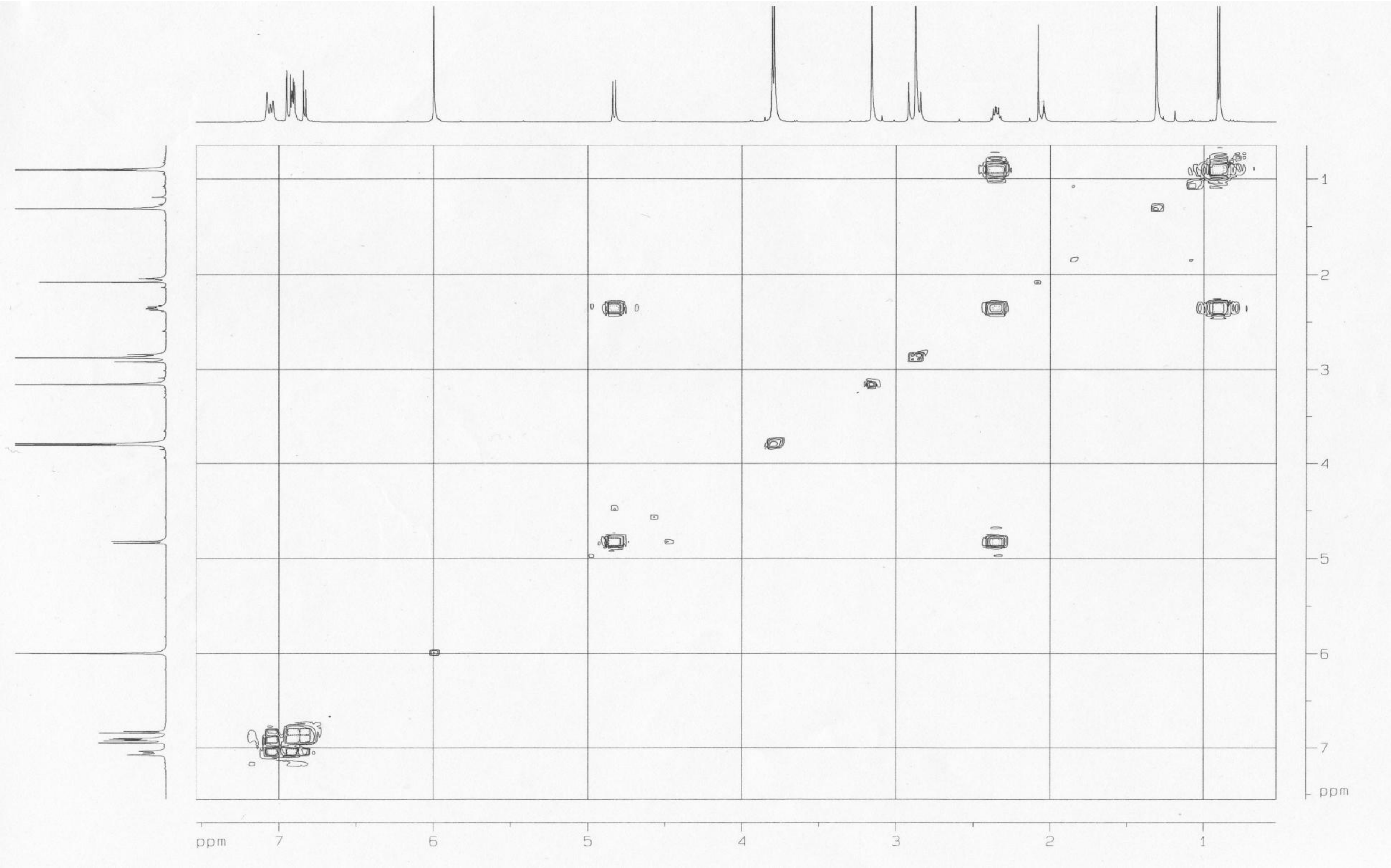

Figure S14. HMBC spectrum of **2**

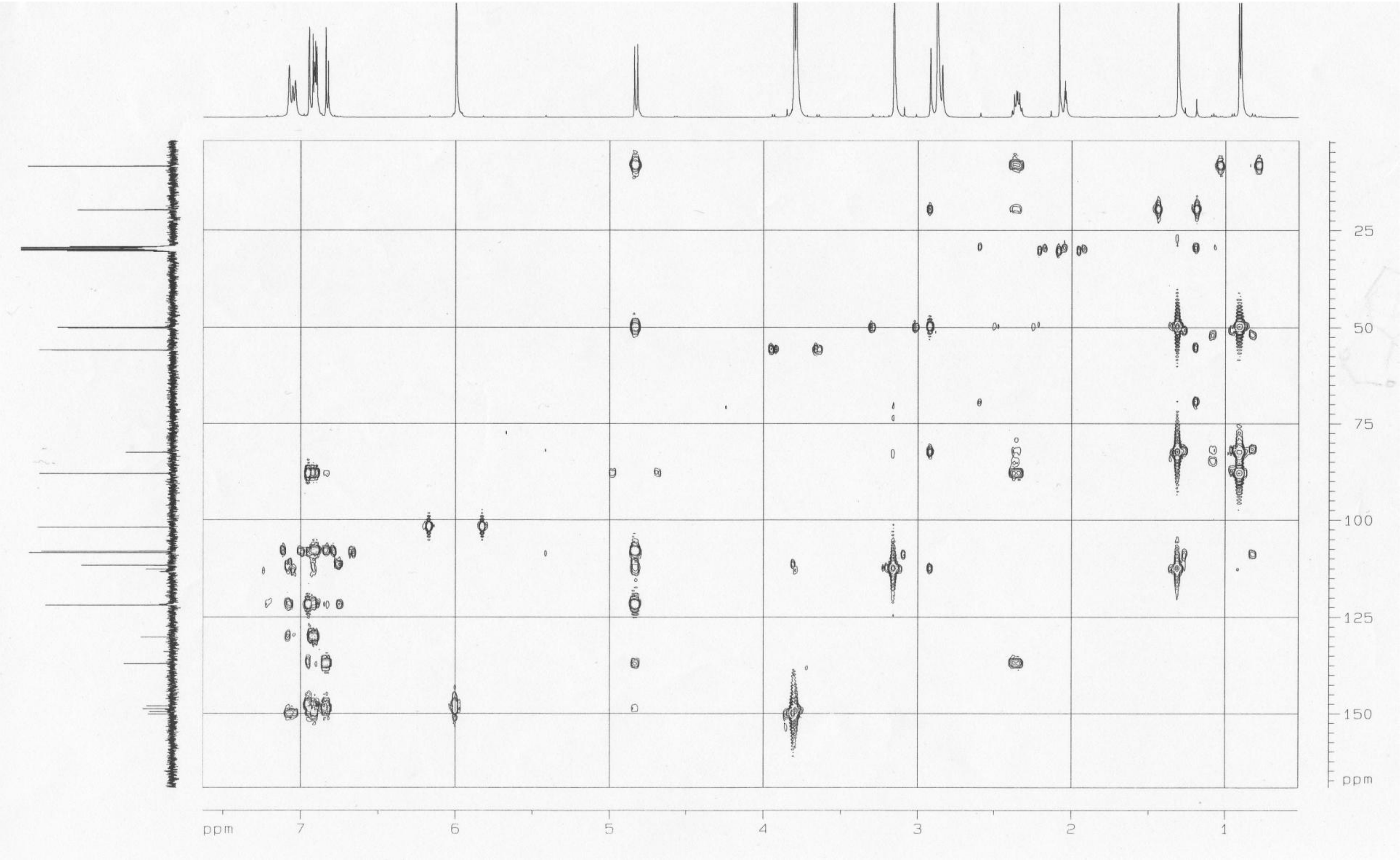

Figure S15. ROESY spectrum of **2**

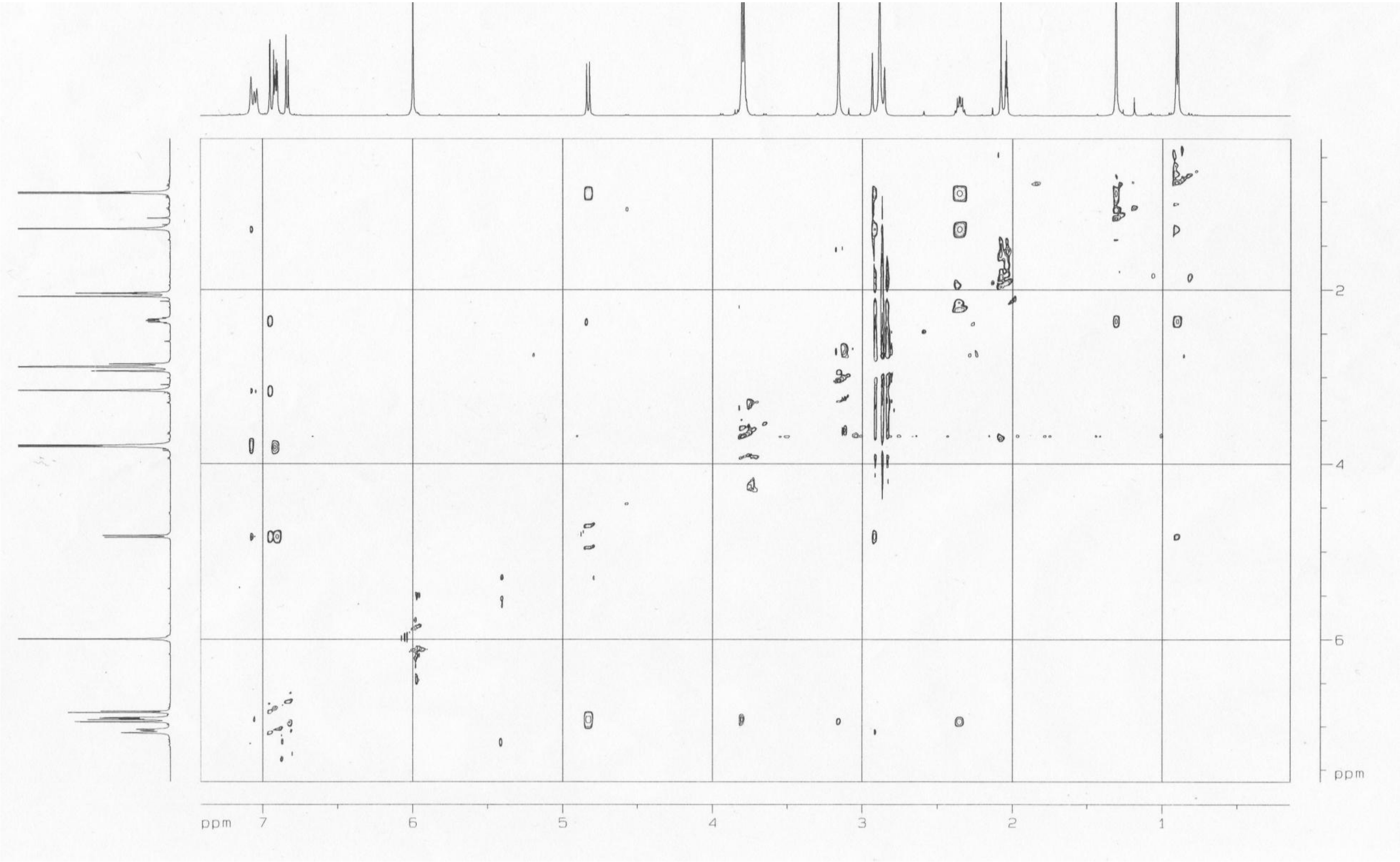

Figure S16. CD spectrum of **2**

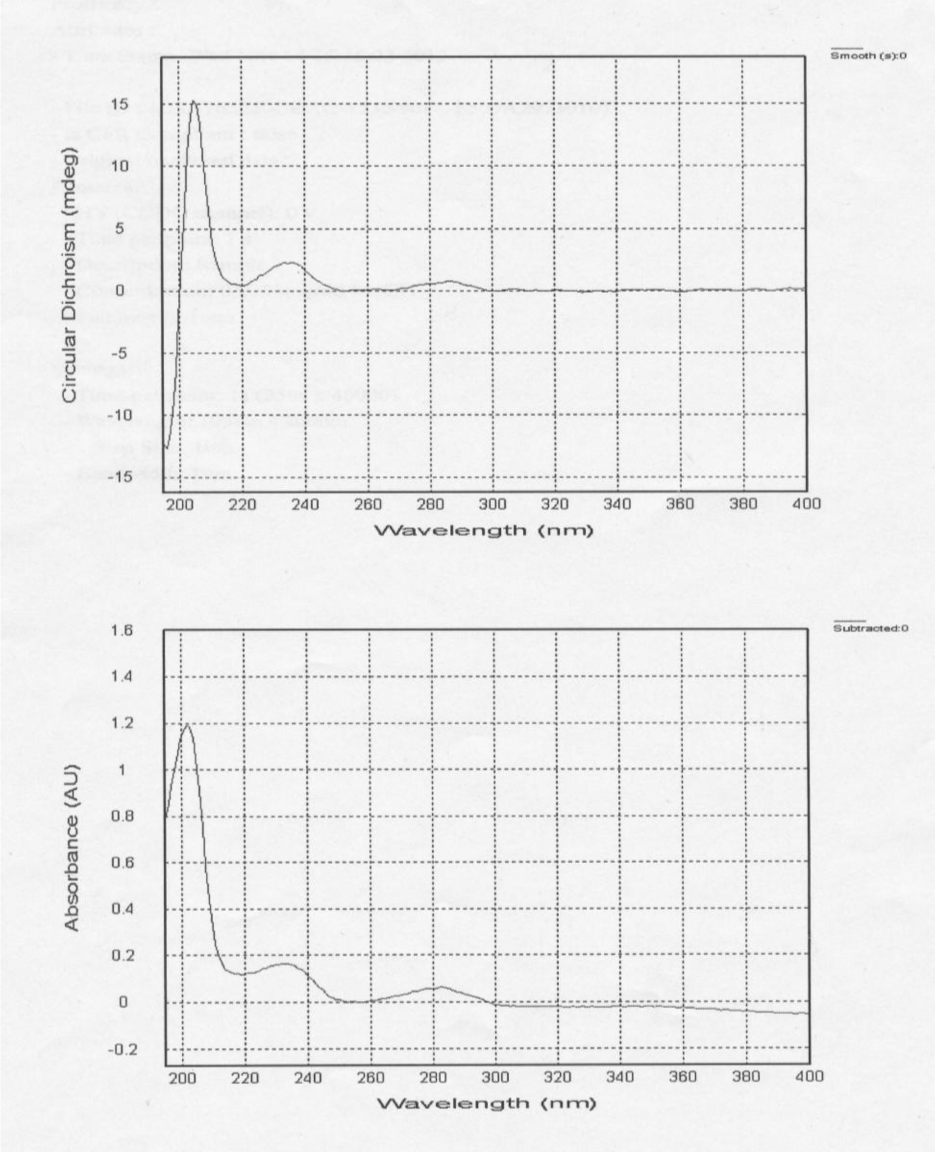

Figure S17. HREI spectrum of **3**

Elemental Composition Report

Page 1

Single Mass Analysis

Tolerance = 10.0 PPM / DBE: min = -10.0, max = 120.0
Selected filters: None

Monoisotopic Mass, Odd and Even Electron Ions
23 formula(e) evaluated with 1 results within limits (up to 51 closest results for each mass)
Elements Used:
C: 0-200 H: 0-400 O: 5-8

sww67
09:32:05 02-Jul-2012
Voltage EI+

KIB
M120702EA-01AFAMMA 19 (1.744)
388.1514

Autospec Premier
P776
79.0

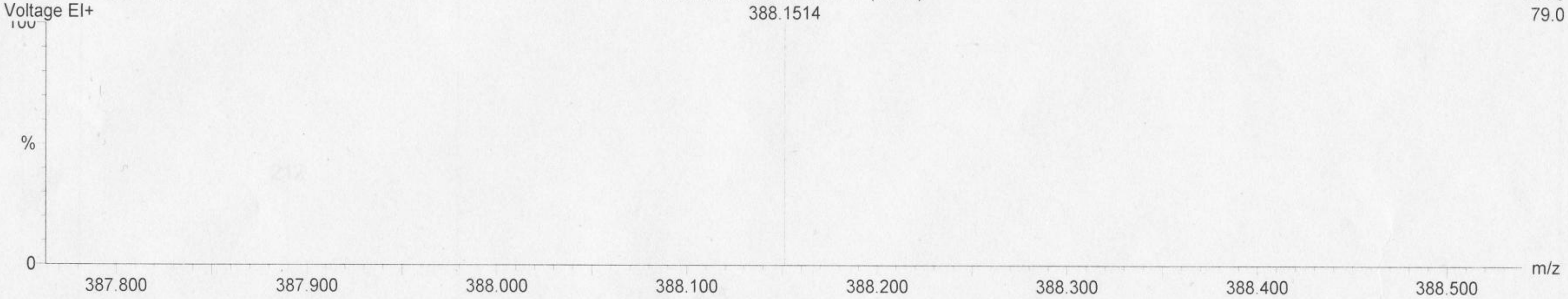

Minimum:				-10.0		
Maximum:		100.0	10.0	120.0		
Mass	Calc. Mass	mDa	PPM	DBE	i-FIT	Formula
388.1514	388.1522	-0.8	-2.1	10.0	5546054.0	C21 H24 O7

Figure S18. ^{1}H NMR spectrum of **3**

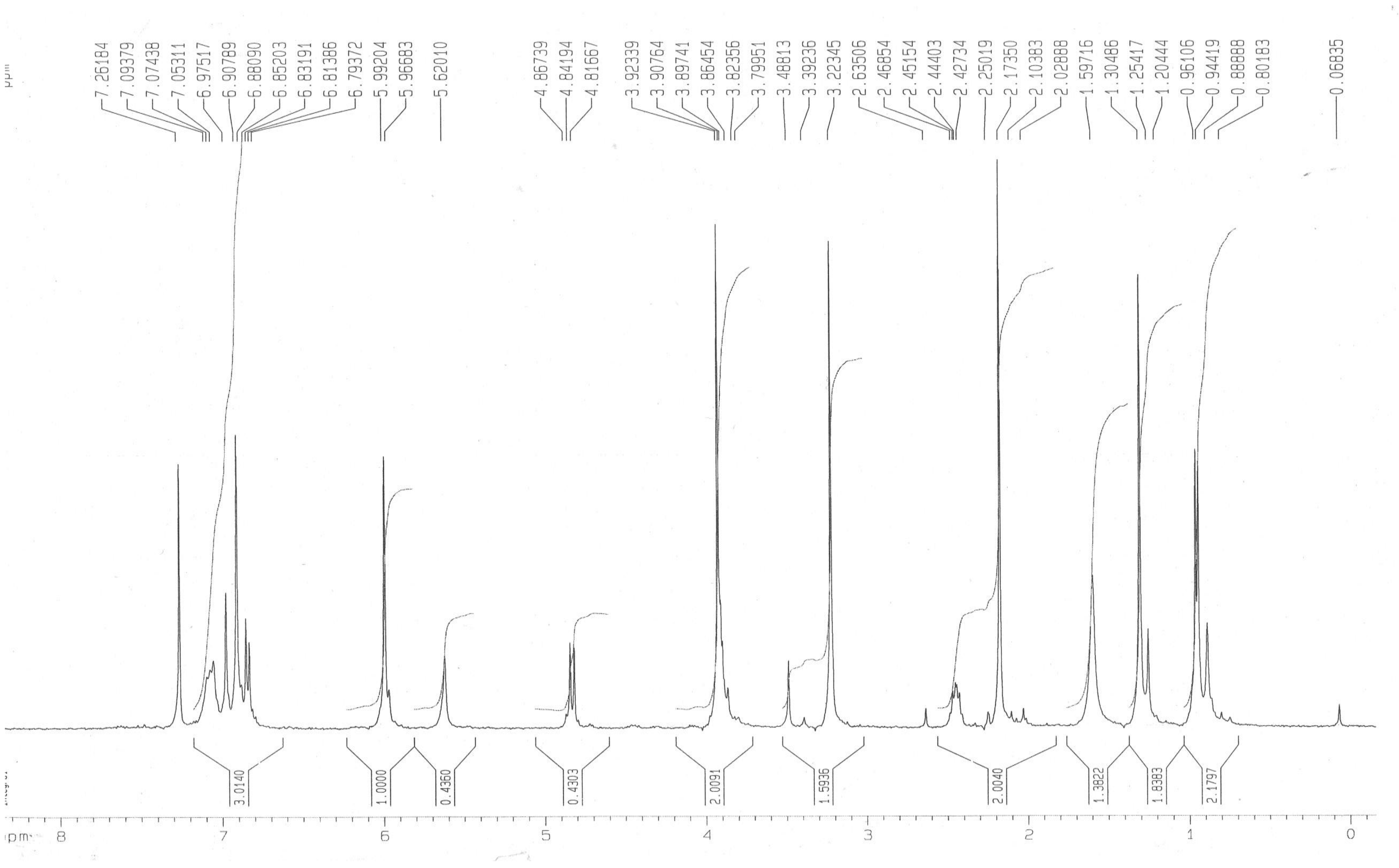

Figure S19. ^{13}C NMR spectrum of **3**

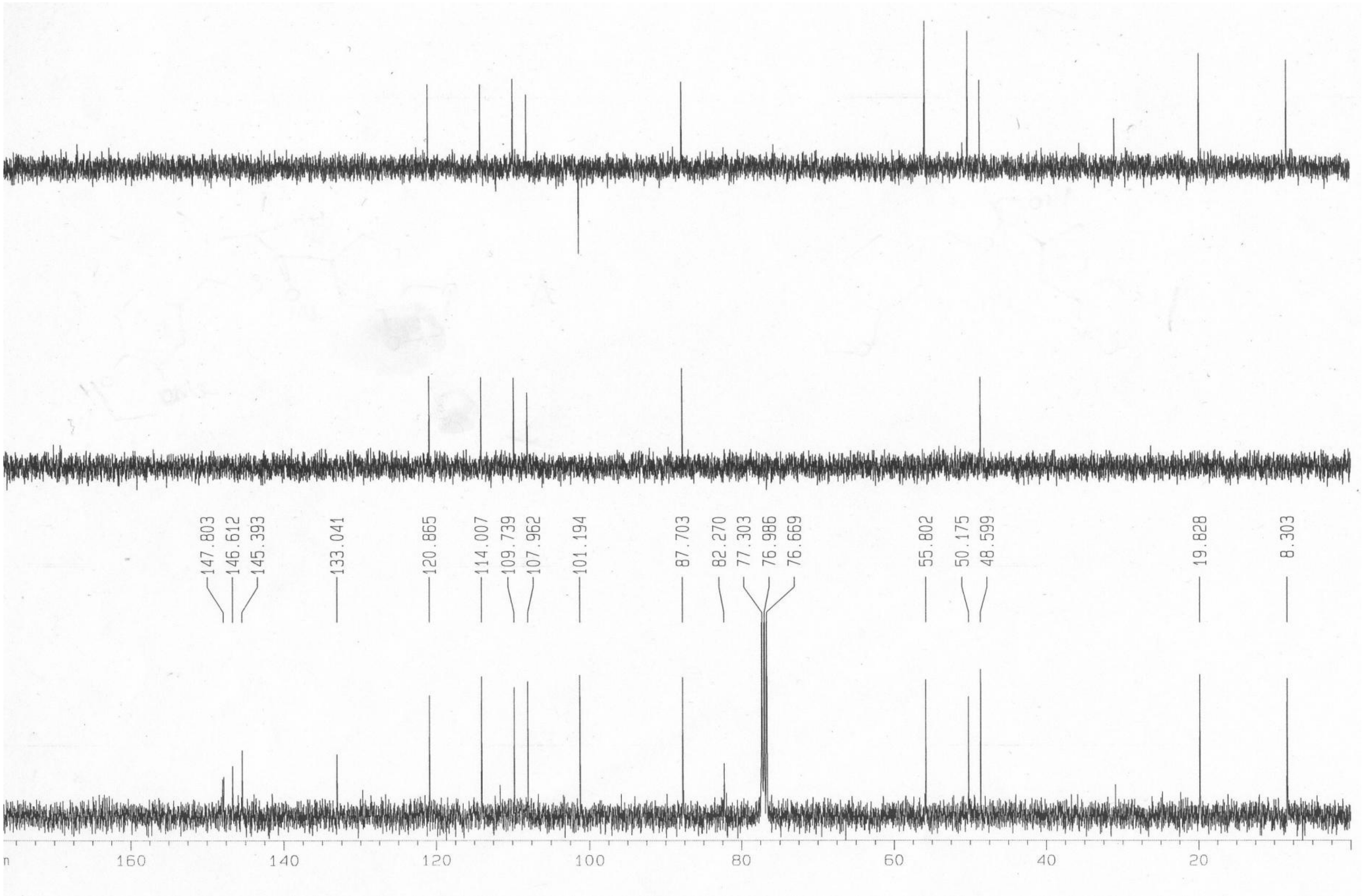

Figure S20. CD spectrum of **3**

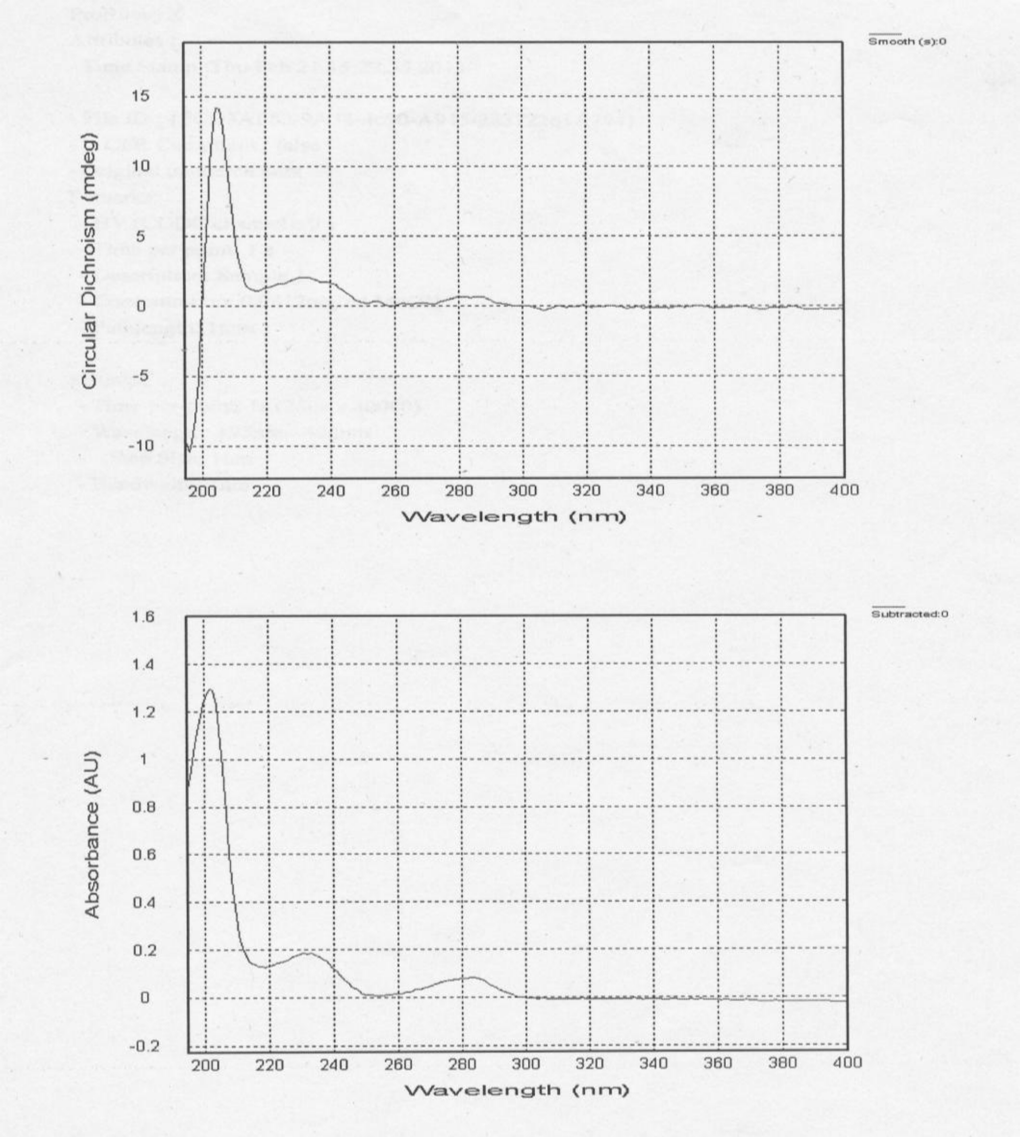

Figure S21. HREI spectrum of **4**

Elemental Composition Report

Page 1

Single Mass Analysis

Tolerance = 10.0 PPM / DBE: min = -10.0, max = 120.0

Selected filters: None

Monoisotopic Mass, Odd and Even Electron Ions

45 formula(e) evaluated with 1 results within limits (up to 51 closest results for each mass)

Elements Used:

C: 0-200 H: 0-400 O: 3-10

sww96
16:12:31 14-Jul-2012
Voltage EI+

KIB
M120713EA-01AFAMM 31 (2.846)

Autospec Premier
P776
29.2

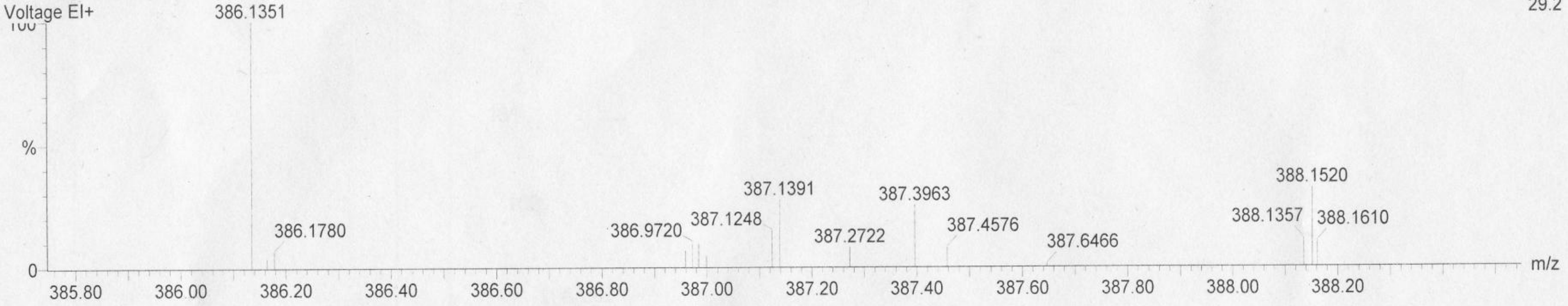

Minimum:				-10.0		
Maximum:		100.0	10.0	120.0		
Mass	Calc. Mass	mDa	PPM	DBE	i-FIT	Formula
388.1520	388.1522	-0.2	-0.5	10.0	5546025.5	C21 H24 O7

Figure S22. ^{1}H NMR spectrum of **4**

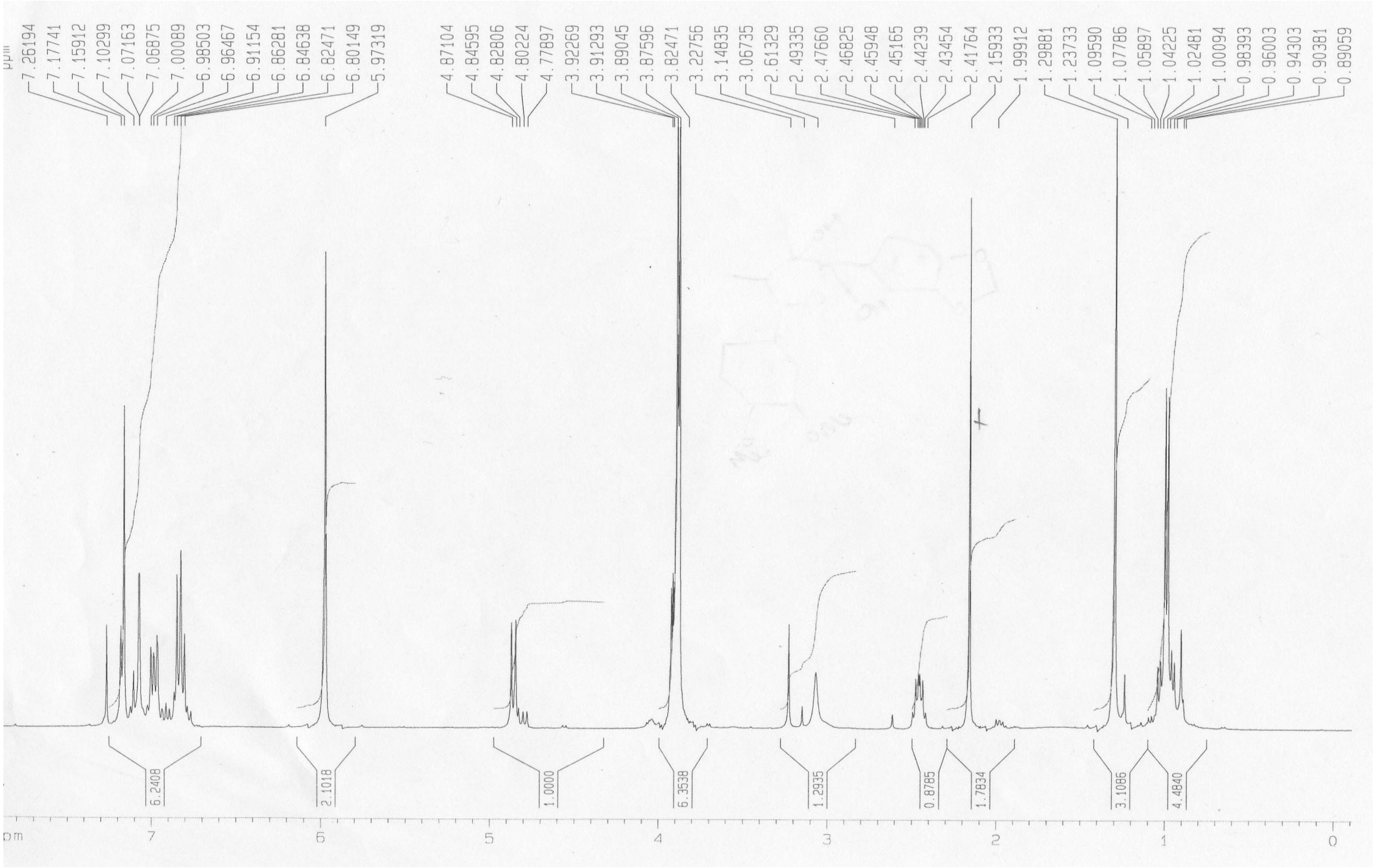

Figure S23. ^{13}C NMR spectrum of **4**

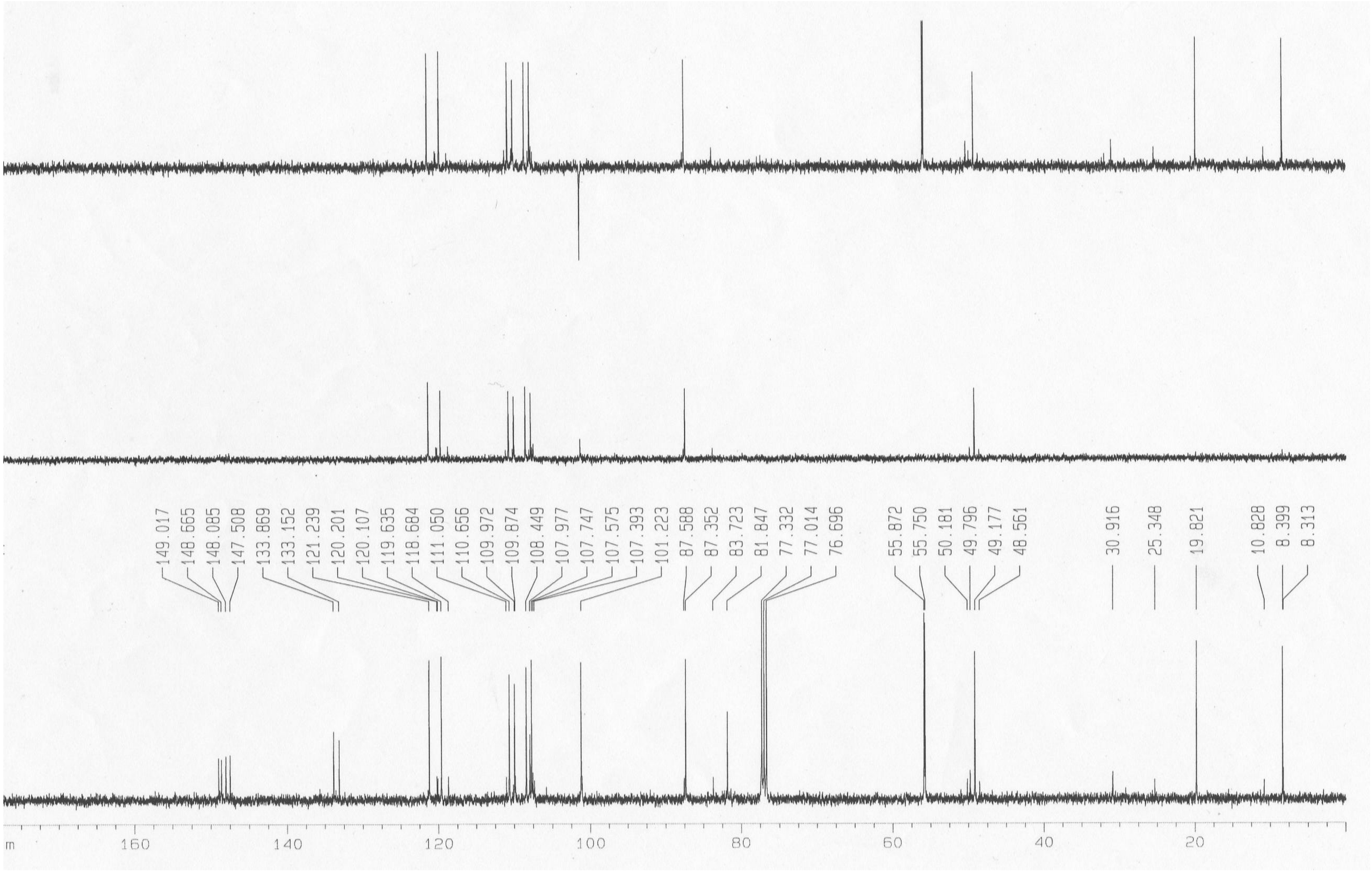

Figure S24. CD spectrum of **4**

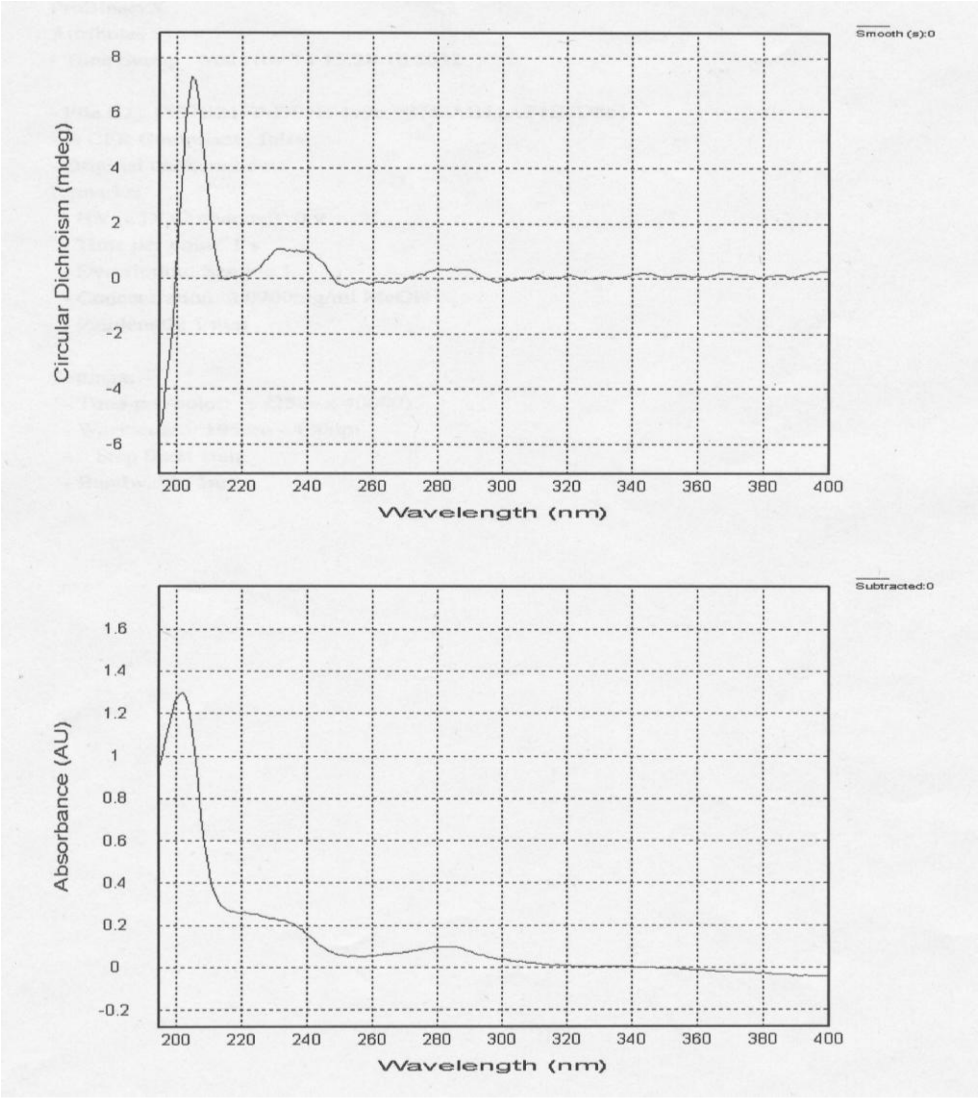

Supplement: Supplementary file 1 — Supplementary material, approximately 4.15 MB. [file 13659_2013_17_MOESM1_ESM.pdf]
